# *Dipeptidyl peptidase III* as a DNA marker to investigate epidemiology and taxonomy of Old World *Leishmania* species

**DOI:** 10.1371/journal.pntd.0009530

**Published:** 2021-07-26

**Authors:** Insaf Bel Hadj Ali, Hamed Chouaieb, Yusr Saadi Ben Aoun, Emna Harigua-Souiai, Hejer Souguir, Alia Yaacoub, Oussaïma El Dbouni, Zoubir Harrat, Maowia M. Mukhtar, Moncef Ben Said, Nabil Haddad, Akila Fathallah-Mili, Ikram Guizani

**Affiliations:** 1 Laboratory of Molecular Epidemiology and Experimental Pathology, Institut Pasteur de Tunis, Université de Tunis El Manar, Tunisia; 2 Service de parasitologie, EPS Farhat Hached, Faculté de Médecine de Sousse, Université de Sousse, Sousse, Tunisia; 3 Department of Infectious Diseases, Rafik Hariri Hospital, Beirut, Lebanon; 4 Laboratoire d’Eco-épidémiologie Parasitaire et Génétique des Populations, Institut Pasteur d’Algérie, Algiers, Algeria; 5 Bioscience Research Institute, Ibn Sina University, Khartoum, Sudan; 6 Laboratory of Immunology and Vector-Borne Diseases, Faculty of Public Health Lebanese University, Hadath, Lebanon; U.S. Food and Drug Administration and Center for Biologics Evaluation and Research, UNITED STATES

## Abstract

**Background:**

*Dipeptidyl peptidase III* (*DPPIII*) member of M49 peptidase family is a zinc-dependent metallopeptidase that cleaves dipeptides sequentially from the N-terminus of its substrates. In *Leishmania*, *DPPIII*, was reported with other peptidases to play a significant role in parasites’ growth and survival. In a previous study, we used a coding sequence annotated as *DPPIII* to develop and evaluate a PCR assay that is specific to dermotropic Old World (OW) *Leishmania* species. Thus, our objective was to further assess use of this gene for *Leishmania* species identification and for phylogeny, and thus for diagnostic and molecular epidemiology studies of Old World *Leishmania* species.

**Methodology:**

Orthologous *DDPIII* genes were searched in all *Leishmania* genomes and aligned to design PCR primers and identify relevant restriction enzymes. A PCR assays was developed and seventy-two *Leishmania* fragment sequences were analyzed using MEGA X genetics software to infer evolution and phylogenetic relationships of studied species and strains. A PCR-RFLP scheme was also designed and tested on 58 OW *Leishmania* strains belonging to 8 *Leishmania* species and evaluated on 75 human clinical skin samples.

**Findings:**

Sequence analysis showed 478 variable sites (302 being parsimony informative). Test of natural selection (dN-dS) (-0.164, SE = 0.013) inferred a negative selection, characteristic of essential genes, corroborating the *DPPIII* importance for parasite survival. Inter- and intra-specific genetic diversity was used to develop universal amplification of a 662bp fragment. Sequence analyses and phylogenies confirmed occurrence of 6 clusters congruent to *L*. *major*, *L*. *tropica*, *L*. *aethiopica*, *L*. *arabica*, *L*. *turanica*, *L*. *tarentolae* species, and one to the *L*. *infantum* and *L*. *donovani* species complex.

A PCR-RFLP algorithm for *Leishmania* species identification was designed using double digestions with *Hae*III and *Kpn*I and with *Sac*I and *Pvu*II endonucleases. Overall, this PCR-RFLP yielded distinct profiles for each of the species *L*. *major*, *L*. *tropica*, *L*. *aethiopica*, *L*. *arabica* and *L*. *turanica* and the *L*. (*Sauroleishmania) L*. *tarentolae*. The species *L*. *donovani*, and *L*. *infantum* shared the same profile except for strains of Indian origin. When tested on clinical samples, the *DPPIII* PCR showed sensitivities of 82.22% when compared to direct examination and was able to identify 84.78% of the positive samples.

**Conclusion:**

The study demonstrates that *DPPIII* gene is suitable to detect and identify *Leishmania* species and to complement other molecular methods for leishmaniases diagnosis and epidemiology. Thus, it can contribute to evidence-based disease control and surveillance.

## Author summary

Leishmaniases correspond to a group of neglected tropical diseases caused by protozoan parasites members of more than 20 *Leishmania* species. Leishmaniases are epidemiologically complex diseases. Their complexity relates to the diversity of species and transmission cycle components, often poorly elucidated. Risk factors for changing eco-epidemiological profiles include environmental and climate changes, migrations and conflicts, contributing to leishmaniases emergence in disease-free areas, leading to co-sympatry of the major pathogens in the same transmission areas and even foci. Taxonomical identification of the parasites is a central issue for patient management, molecular epidemiology and thus disease control and surveillance. Thus, development of adequate specific and sensitive DNA tools to identify and classify *Leishmania* parasites for surveillance and control remains a research priority. The *DPPIII* gene is an essential gene conserved in all *Leishmania* species. By comparative sequence analyses of the *DPPIII* gene fragments and the development of *DPPIII*-based PCR RFLP assays, we showed its potential for molecular diagnosis, epidemiology and taxonomy. We conclude that this molecular marker can be used to complement other molecular methods for OW *Leishmania* species identification and discrimination and can contribute to evidence- based disease control and surveillance.

## Introduction

Leishmaniases correspond to a group of diseases caused by more than 20 protozoan *Leishmania* species parasites. They are transmitted to humans by the bites of infected female phlebotomine sandflies. Three main disease forms are encountered: cutaneous, visceral and mucocutaneous leishmaniases. An estimated 1 to 1.5 million cutaneous leishmaniasis (CL) and 500000 visceral leishmaniasis (VL) cases are annually reported worldwide [1,2]. Species identification of *Leishmania* is crucial for selecting the most appropriate therapy to be administrated to patients [3–5]. Parasite classification was initially based on eco-biological criteria including vectors, geographical distribution, clinical manifestation and disease tropism [6–9]. But nowadays, it is based on molecular technologies [10]. Leishmaniases are considered as taxonomically and epidemiologically complex diseases. Their complexity relates to the co-endemicity of the major pathogens [11,12], the diversity of transmission cycle components, often poorly elucidated, environmental and climate changes [13,14], migrations and conflicts [15], all contributing to leishmaniases emergence in disease-free areas [16] and potential involvement of novel hosts [17,18].

In Tunisia and worldwide, these diseases show emerging trends in their epidemiology resulting in changes in their distribution illustrated by the spread of the species from their classical foci to neighboring areas [19–24]. Identification of these emerging foci is important for disease control. This is mainly based on the use of adequate DNA tools for identifying and classifying *Leishmania* parasites. Advances in systematics and taxonomy methods have improved knowledge about parasite evolution, phylogeny, and molecular species identification. *Leishmania* species discrimination and evolutionary relationships have been inferred using a range of assays targeting molecular markers including coding regions such as for *heat shock* proteins (*hsp 20* [25] or *hsp70* [26]), *glycoprotein 63* (*gp63*) [27–29], *cytochrome oxidase II* [30,31], *cysteine protease B* [32,33], *Tryparedoxine peroxidase* [34], and non-coding regions such as the internal transcribed spacer (ITS) 1 and 2 [35–37] and some repetitive regions [38]. Some assays are specific to certain taxonomic groups [30], others lack sensitivity [39] or specificity [34], or are only suitable for intra-specific analyses using for instance microsatellites markers [15,40–42]. Increasing knowledge about evolutionary relationships is generated through genome projects [43,44] but also by resorting to well characterized markers that provide thorough supplementary data about inter and intra-species variations in clinical isolates. Such knowledge improves debated phylogeny and taxonomy [43,45–47] and strengthens capacity for molecular epidemiology investigations.

Metallopeptidases constitute a diverse group of distinct evolutionary families. *Dipeptidyl peptidase III* (*DPPIII*) belongs to the M49 peptidase family of zinc-dependent enzymes that sequentially cleave dipeptides from the N-terminus of its substrates [48]. Studies suggested the involvement of the human *DPPIII* protein in protein turn over, oxidative stress and pain modulation and inflammation [49]. In *Leishmania*, the protein was characterized in *L*. *braziliensis* demonstrating its role in parasite survival in the vector, or host environments through peptide degradation and thus use of amino acids for energy production in stressful environment [50]. Presence of this protein was demonstrated in the secretome of *L*. *donovani*, which classified it as a candidate virulence factor that might be part of a stress response of the parasite [51]. A 2040bp *DPPIII* gene is present in all *Leishmania* and other trypanosomatids sequenced genomes [50]. However, the gene seemed absent in the *Trypanosoma* genus including *T*. *brucei* and *T*. *cruzi* species [50,52]. The *L*. *braziliensis DPPIII* translated sequence has a 65–80% similarity rate to other microorganisms belonging to *Endotrypanum*, *Blechomonas*, *Crithidia* and *Leptomonas* genera [50]. Within *Leishmania*, the gene is conserved in species infecting humans and other mammals, with predicted 88–89% amino acids sequence identity to the *L*. *braziliensis* enzyme. In a previous study, primers targeting one part of the gene were used to amplify *L*. *major*, *L*. *tropica*, *L*. *arabica* and *L*. *aethiopica* DNAs but the reaction did not yield any product with the viscerotropic *L*. *infantum* and *L*. *donovani* species DNAs nor with *L*. *tarentolae* [53]. In this study, we aimed at extending the validation of this gene as a potential target for DNA diagnosis of Old World *Leishmania* species including in countries which are also endemic for viscerotropic species. Therefore, we further investigated genetic diversity of the *DPPIII* gene and products of a new PCR in a panel of strains belonging to a range of pathogenic and non- pathogenic endemic species in the Old World to assess its relevance as a molecular marker for phylogeny and species identification, and thus to complement others for molecular epidemiology studies of Old World *Leishmania* species and disease surveillance.

## Methods

### Ethical statement

This work complies with the ethical standards as required by the ethical committee of Institut Pasteur de Tunis, lead institution in the studies (Ref. 2016/24/I/LRIPT04; 2016/13D/I/CIC).

The samples correspond to scrapings done in the frame of routine diagnosis of suspected CL patients with consent of patients in prospective studies (samples 1–13). In case of samples 14 to 75, they were also collected in the frame of routine diagnosis of suspected patients, the preserved samples’ remains were used retrospectively with the consent of the ethics committee of IPT with respect of patient anonymity.

### Parasite strains and DNAs

In total, we analyzed 58 *Leishmania* DNA belonging to 8 *Leishmania* species isolated from a range of hosts: human cases, reservoirs or vectors, in various geographical origins, to cover a maximum of Old-World parasite species, available in our local DNA bank as detailed in S1 Table. They correspond to already characterized strains or DNAs by isoenzyme, and genomic RFLP [54] or PCR-RFLP ITS1 [55], obtained from reference centers in Montpellier or Rome, and in case of clinical isolates from health centers in Tunisia, Sudan and Algeria (S1 Table). The promastigote DNAs were extracted by phenol-chloroform procedure as described [54].

### Human CL samples

Seventy-five DNAs extracted from clinical samples using DNA purification kits as recommended by the supplier (Qiagen QIAamp DNA Mini Kit) were used (S2 Table). The samples correspond to scrapings done in the frame of routine diagnosis of suspected CL patients with consent of patients in prospective studies (samples 1–13) in clinical departments in Tunisia (N = 10; University Hospital Farhat Hached, Sousse) and Lebanon (N = 3; Rafik Hariri Hospital, Beirut). In case of remains of lesion scrapings made during diagnosis at the clinical parasitology department of the Farhat Hached University hospital in Sousse, Tunisia (N = 62; samples 14–75), they were used with the consent of the ethics committee of IPT. This sampling includes CL cases and patients having other cutaneous/ dermal diseases.

CL was confirmed for 45 patients by direct examination (DE) under microscope (x1000) of Giemsa- stained smears and subsequent observation of amastigotes. Twenty- six were DE negative, and in case of 4 CL patients, the direct examination was not done or not reported on the data collection file. For molecular identification purposes, ITS1-PCR followed by *Hae*III RFLP was used as an identification technique as previously described [55]. No- template reactions were the internal negative controls of the assays; positive reaction controls contained known *Leishmania* DNAs inputs that were at the limit of detection.

Performances of ITS1 PCR and *DPPIII* PCR assays were computed using DE as gold standard. Besides sensitivity and specificity, we generated the receiver operating curves (ROC) using the scikit-learn library under Python3. The area under curve (AUC) was calculated to estimate the ability of a given diagnosis test to correctly differentiate positive *versus* negative cases.

### PCR design and analyses of the products

**Fig 1:**
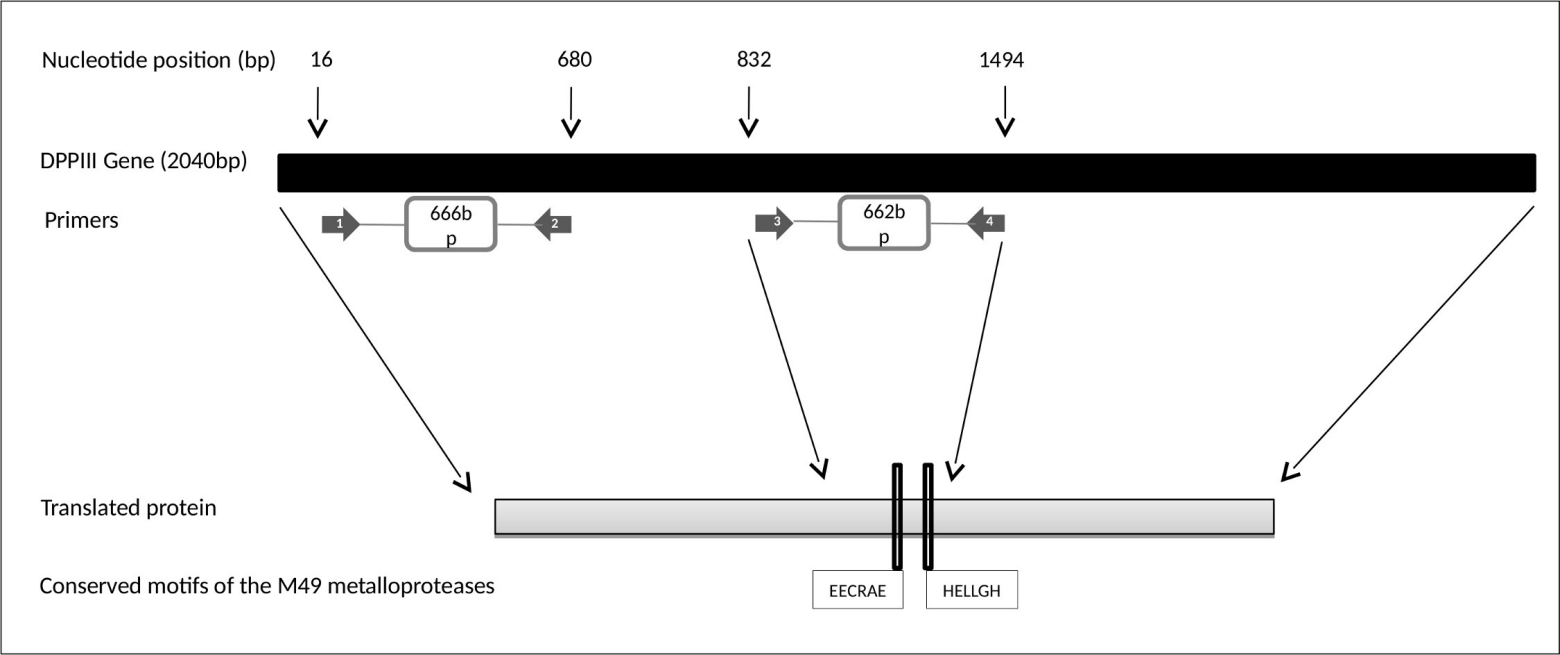
Schematic representation of the *DPPIII* gene and PCR primers positions. 1 & 2: Primers used by [53] (DPP F/DPP R). 3 & 4: Primers designed in this study (*DPPIII*-F/*DPPIII*-R) amplify a 662bp fragment.

Available *dipeptidyl peptidase III* gene sequences of different *Leishmania* species (S3 Table) were retrieved from TriTrypDB Release 26 (14 Oct 15) and aligned using Geneious v3.6.2 computer program to investigate sequence polymorphisms including in restriction sites and design PCR based assays. Restriction sites having minimum effective recognition sequence length of 4 nucleotides were searched within the Restriction Enzyme Database (REBASE, http://rebase.neb.com/). Two generic primers were manually designed in a gene region selected upon this multiple alignment analysis in conserved parts of the gene (Fig 1) flanking a region showing extensive inter-species polymorphisms. Then to verify adequacy of this design, the primers were further analyzed and their sequence was adjusted for primer melting temperature, primer secondary structures including hairpins, self-dimers, and occurrence of cross-dimers in the primers pair using NetPrimer, an online primer analysis software (http://www.premierbiosoft.com/netprimer/). NCBI primer-blast analysis did not find a target template in *Homo sapiens* genome database. Finally, the retained primers cover the region of the gene defined by the positions 832 to 1494 and have the following sequences: *DPPIII*-F 5’-AGGAGTGGGTGAAGGATGTG-3’ and *DPPIII*-R 5’-CAGCAAGCAGAGGTACAGC-3’.

The reaction was set up to amplify 0.2 ng of DNA in 25μL using a mix that contained 1x standard PCR buffer, 1.5 mM MgCl_2_, 200 μM of each 2’- deoxy-nucleoside 5’-triphosphate, 1U Taq Platinium DNA polymerase (Invitrogen), 0.3 μM of each primer. No template reactions were the negative PCR controls. The thermal cycling parameters of the assay were as follows: initial denaturation at 94°C for 3 min followed by 35 cycles consisting of 94°C for 1 min, 62°C for 2 min, and 72°C for 2 min, and a final extension step of 5 min at 72°C. Amplicons were visualized on a 1.5% agarose gel under UV light in presence of ethidium bromide.

Analytical detection limit of the assay was assessed testing serial ten-fold DNA dilutions, ranging from 20ng/μl to 0.002fg/μl, whereby 1μl was used for PCR.

The *DPPIII* F/R PCR products were also sequenced or digested using selected enzymes.

Direct sequencing of PCR products was performed on both strands using the *DPPIII*-F and *DPPIII*-R oligonucleotides, the BigDye Terminator v3.1 Cycle sequencing Kit (Applied Biosystem), and an ABI 3500 sequencer. The chromatograms were visualized and manually adjusted using DNA Baser sequence assembler v4 program (2013) (Heracle Biosoft, www.DnaBaser.com).

In case of RFLP analysis, the enzymes were first selected for their ability to differentiate OW *Leishmania* species with a minimum number of cuts and without generating too small fragments (<50bp) using predicted RFLP patterns visualized with the SnapGene 2.8.3 program from GSL Biotech, available at snapgene.com. Double digestions were done in 20μl reactions using 1 unit of each restriction enzyme, *Hae*III and *Kpn*I or *Sac*I and *Pvu*II, as recommended by the manufacturer (New England Biolabs *Inc*.) using 1x CutSmart buffer, and 10μl of reaction product. Incubations were done at 37°C for 2h. Then the restriction fragments were visualized on a 3% agarose gel.

### Phylogenetic analyses

Phylogenetic analysis was foremost carried out with previously published sequences (S3 Table) with the software package MEGAX (Molecular Evolutionary Genetic Analysis across computing platforms [56]). Nineteen sequences corresponding to 16 *Leishmania* (representing 13 *Leishmania* species), one *L*. (*Sauroleishmania*) and two *Leptomonas* species (*Leptomonas pyrrhocoris* and *Leptomonas seymouri*) were aligned using ClustalW program, and MEGAX software was used to build phylogenetic trees. Then, sequenced *DPPIII* fragments of a selection of DNAs (S1 Table) were aligned with the published sequences for further phylogenetic analyses of the amplified fragment.

Evolutionary distances were computed with the substitution model Tamura 3-parameter method [57] with Gamma distribution variation rate [58] (shape parameter = 0.65) chosen using the Bayesian information criterion (BIC), as implemented in the MEGAX software. Monophyletic groups were supported by 1000 bootstrap resampling method [59]. UPGMA [60], Neighbor-Joining (NJ) [61], Minimum Evolution (ME) [62], and Maximum likelihood (ML) [63] phylogenetic trees were constructed as implemented in MEGAX software.

We also used MEGAX to analyze the extent of sequence variation by calculating the number of polymorphic and parsimony-informative sites. The number of synonymous substitutions by synonymous sites and non-synonymous substitutions by non-synonymous sites were calculated from averaging the distance estimation of the codon-based evolutionary divergence over all sequence pairs using Nei-Gojobori model [64] from which the subsequent difference between dN and dS was deduced. Standard errors were obtained by a bootstrap procedure (1000 replicates). We used codon-based Z-test of neutral evolution to assess if the gene is under a selective pressure. Average sequence composition and the average percentage of pairwise similarity over the alignment were determined using the Geneious 3.6.2 program.

## Results

### In silico analysis of the entire gene confirms taxonomical potential of DPPIII gene

We used phylogenetic analysis to assess taxonomical potential of the *DPPIII* gene and to detect species- specific single nucleotide polymorphisms (SNPs) and indels. We did multiple sequence alignments using the entire *DPPIII* gene (2040bp) of 19 published database sequences, covering 13 *Leishmania*, 1 *L*. (*Sauroleishmania*) and 2 *Leptomonas* species (S3 Table). Sequence analysis revealed 478 variable sites of which 302 (63%) were parsimony informative (S4 Table). To determine the selective evolutionary pressure on the predicted *DPPIII* protein, codon-based evolutionary divergence from averaging all sequence pairs was used to estimate the number of synonymous substitutions per synonymous site (dS = 0.199; SE = 0.003) and the number of non-synonymous substitutions per non-synonymous sites (dN = 0.035; SE = 0.012). The deduced difference between the non-synonymous and synonymous distances per site from averaging over all sequence pairs dN-dS is -0.164 (SE = 0.013). The probability of rejection of the null hypothesis of strict neutrality (dN = dS) using the codon- based Z test of neutrality was in favor of the deviation of the *DPPIII* gene from neutrality and tendency for a negative selection (dN<dS; p<0.05).

**Fig 2:**
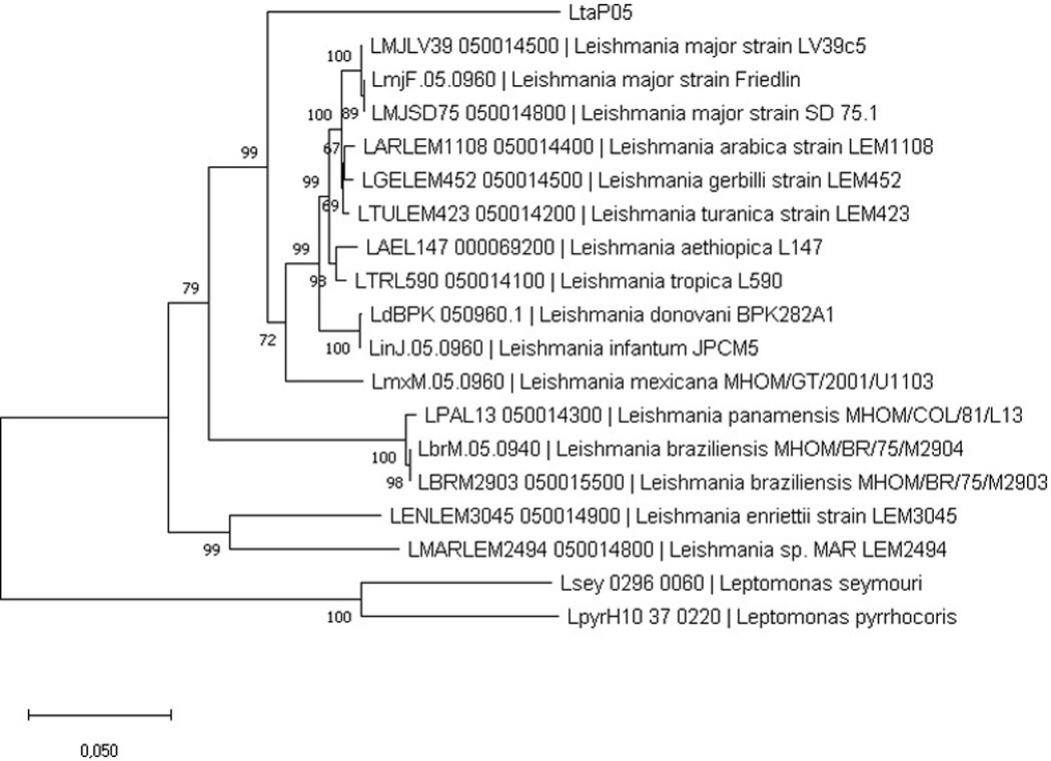
Maximum Likelihood tree constructed using published database kinetoplastid sequences of the entire *DPPIII* gene. The evolutionary history of *Leishmania* parasites was inferred using the Maximum Likelihood method and Tamura-Nei model. The tree with the highest log likelihood (-8661.86) is shown. Initial tree(s) for the heuristic search were obtained automatically by applying Neighbor-Join and BioNJ algorithms to a matrix of pairwise distances estimated using the Tamura-Nei model, and then selecting the topology with superior log likelihood value. A discrete Gamma distribution was used to model evolutionary rate differences among sites (5 categories (+G, parameter = 0.4323)). The tree is drawn to scale, with branch lengths measured in the number of substitutions per site. This analysis involved 19 nucleotide sequences. Codon positions included were 1st+2nd+3rd+Noncoding. There were a total of 2046 positions in the final dataset. Evolutionary analyses were conducted in MEGA X.

The topology of the Neighbor joining phylogenetic tree of these sequences matched taxonomy and known evolutionary relationships of the pertaining species. This corroborated the hypothesis regarding the phylogenetic and taxonomic potential of the *DPPIII* gene (Fig 2).

### A valid generic PCR test and its analytic limit of detection

Then, to determine a smaller similarly informative part of the gene that could be used in molecular assays development, we looked at the mutations and restriction sites distribution as markers of species divergence. We selected a variable part of the gene showing 32.2% single nucleotide polymorphisms (SNPs) including variable restriction sites, that also encodes for the active site motives, HELLGH and EECRAE, which are involved in zinc binding. This region was flanked by interspecies conserved sequences that we used to design a novel primers pair, *DPPIII*-F/*DPPIII*-R, for the generic amplification of this part of the gene (Fig 1). It is where the most discriminatory restriction sites were located.

**Fig 3:**
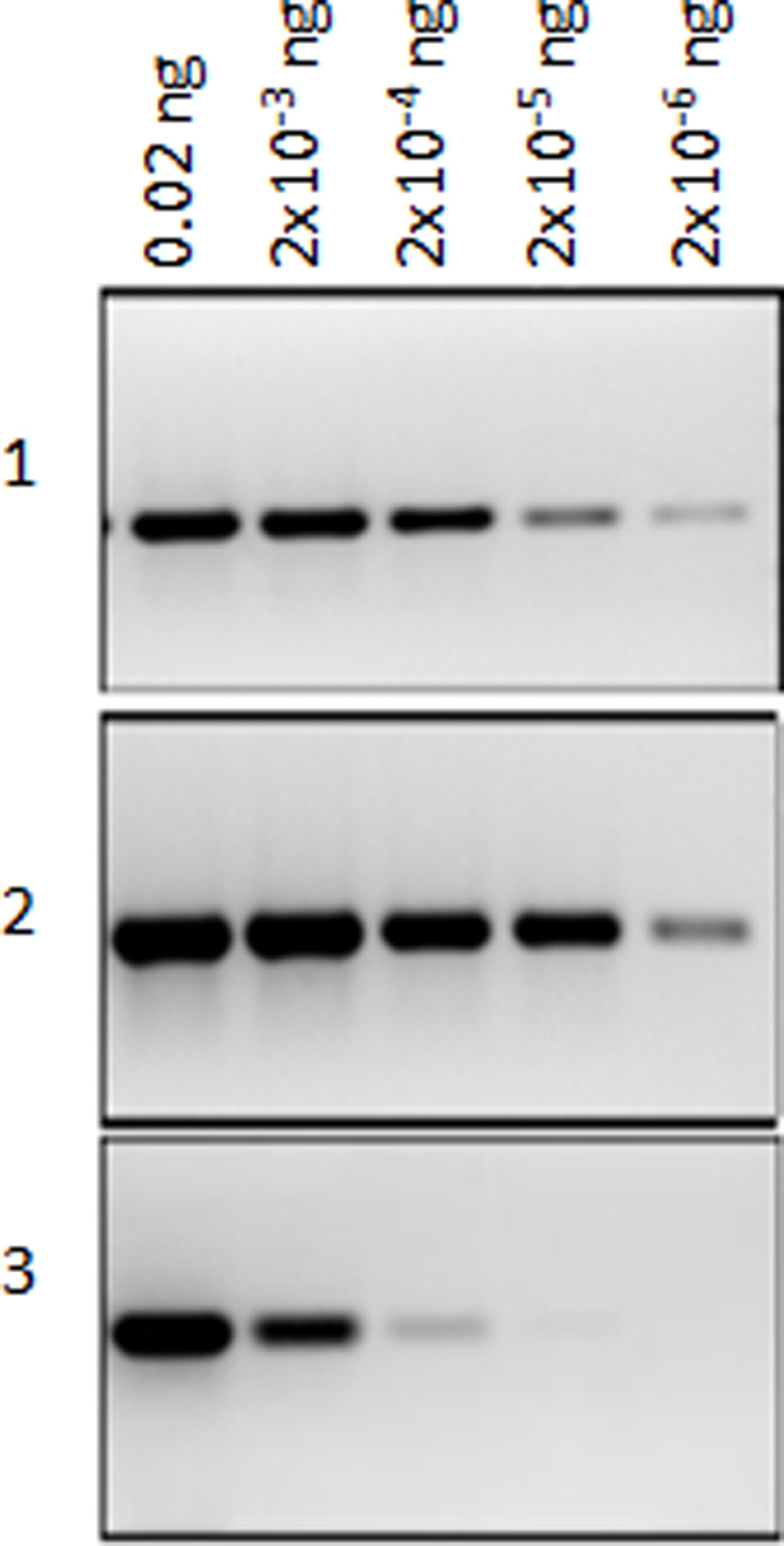
PCR-*DPPIII* detection limit based on ten-fold serial DNA dilutions. **1**: *L*. *major* (Ron44); **2**: *L*. *infantum* (IPT1); **3**: *L*. *tropica* (Bag17). For each DNA, ten- fold serial DNA dilutions starting from 0.02ng were input into the reaction.

The primers amplified the expected 662bp fragment as tested on a representative DNA panel corresponding to OW *Leishmania* and *L*. (*Sauroleishmania*) species, having different geographical origins and hosts (S1 Table), which allowed their validation as generic primers (S1 Fig). In order to assess the analytical sensitivity of this PCR, we tested ten-fold serial DNAs dilutions ranging from 20ng/μl to 0.2fg/μl of three strains (IPT1, Ron44 and Bag17) representing *L*. *infantum*, *L*. *major* and *L*. *tropica* species respectively. The Fig 3 illustrates that depending on the species studied, analytical detection limit varied according to the species/ strain in the 200 – 2fg range. Assuming an average diploid genome mass of 80fg [65], the range of the analytic detection limit was 0.025–2,5 parasites.

### Phylogenetic validation of the amplified 662bp DPPIII gene fragment

To further validate taxonomical potential of the selected 662bp *DPPIII* gene fragment and to possibly deduce species- specific DNA signatures, we successfully sequenced the amplicons of 53 *Leishmania* DNA representing different species and host or geographical origins (S1 Table). In addition, 16 *Leishmania*, 2 *Leptomonas* and 1 *L*. *(Sauroleishmania) DPPIII* sequences were retrieved from the TritrypDB database and included in the analysis (S3 Table). This amounts the sample used in this analysis to a total of 72 sequences of different genera, species and strains. Sequence alignment illustrating the variable sites and the associated haplotypes of all these DNA fragments are shown in S4 and S5 Tables. The sequence analyses of the studied *DPPIII* region among *Leishmania* species revealed the average nucleotide frequencies: T (18.1%), C (28%), A (20.7%), G (33.2%); indels (0.5%); GC content (61.3%) and the average pairwise similarity (96.3%). The selective evolutionary pressure on the 662 bp *DPPIII* fragment was determined, and it showed that as for the entire gene, the studied fragment is under negative selection (dN-dS = -0.178; SE = 0.014).

Twenty haplotypes were identified based on sequence polymorphisms; to each species tested corresponded at least one haplotype. We observed that some SNPs were only seen in species- or group of species- specific haplotypes, which could correspond to DNA signatures of relevance to taxonomy. In addition, in the case of *L*. *tropica* and *L*. *donovani*, some SNPs appeared as specific to some of their strains, may be linked to the geographical origins (S5 Table).

**Fig 4:**
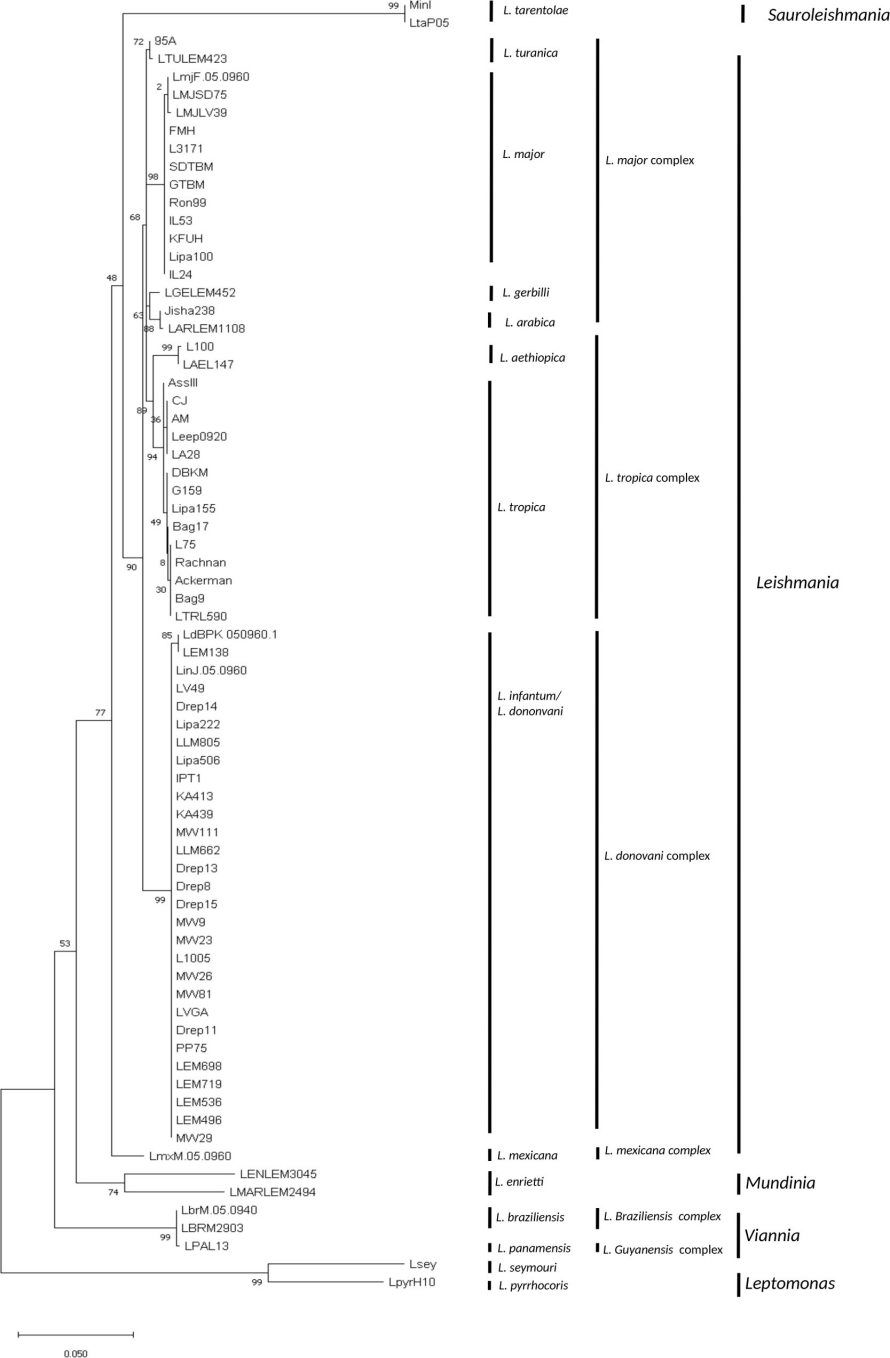
Evolutionary relationships of taxa. The evolutionary history of *Leishmania* parasites was inferred from the 662bp fragment of the *DPPIII* gene using the Maximum Likelihood method and Tamura 3-parameter model. The tree with the highest log likelihood (-2620.35) is shown. The percentage of trees in which the associated taxa clustered together is shown next to the branches. Initial tree(s) for the heuristic search were obtained automatically by applying Neighbor-Join and BioNJ algorithms to a matrix of pairwise distances estimated using the Tamura 3 parameter model, and then selecting the topology with superior log likelihood value. A discrete Gamma distribution was used to model evolutionary rate differences among sites (5 categories (+G, parameter = 0.3096)). The tree is drawn to scale with branch lengths measured in the number of substitutions per site. This analysis involved 72 nucleotide sequences. Codon positions included were 1st+2nd+3rd+Noncoding. There was a total of 662 positions in the final dataset. Evolutionary analyses were conducted in MEGA X.

The Maximum Likelihood based on the amplified *DPPIII* sequence alignment (Fig 4) showed a separation between the New World (*L*. *mexicana*, *L*. *braziliensis*, *L*. *panamensis* and *L*. *enriettii*) and the Old- World species (*L*. *major*, *L*. *tropica*, *L*. *aethiopica*, *L*. *turanica*, *L*. *gerbilli*, *L*. *arabica* and *L*. *infantum/ L*. *donovani*). Among the Old- World species, 6 clusters were observed that are congruent to *L*. *major*, *L*. *tropica*, *L*. *aethiopica*, *L*. *arabica*, *L*. *turanica*, *L*. *tarentolae* species, and another one to the *L*. *infantum* and *L*. *donovani* species complex. This is in accordance with the phylogeny constructed using the entire *DPPIII* gene.

All the groups mentioned above were also observed using UPGMA, NJ and ME phylogenies (S2–S5 Figs) indicating that the derived groups are robust and do not depend on the evolutionary method used.

At the intra-specific level, polymorphisms were observed for different taxa. *L*. *tropica* showed two groups according to their geographical origins. The first group gathers the Mediterranean strains LA28, AM, CJ and Leep0920, and the second, more heterogeneous, includes strains from the Middle East and India. The SNPs that differentiate these subgroups are situated at the positions 1131 and 1350 of the *DPPIII* gene (S5 Table). In the second case, 2 SNPs (1103 and 1187) separated the Indian from the African and Middle Eastern *L*. *donovani* strains that were grouped with the *L*. *infantum* strains (S5 Table).

### PCR-RFLP analysis

**Fig 5:**
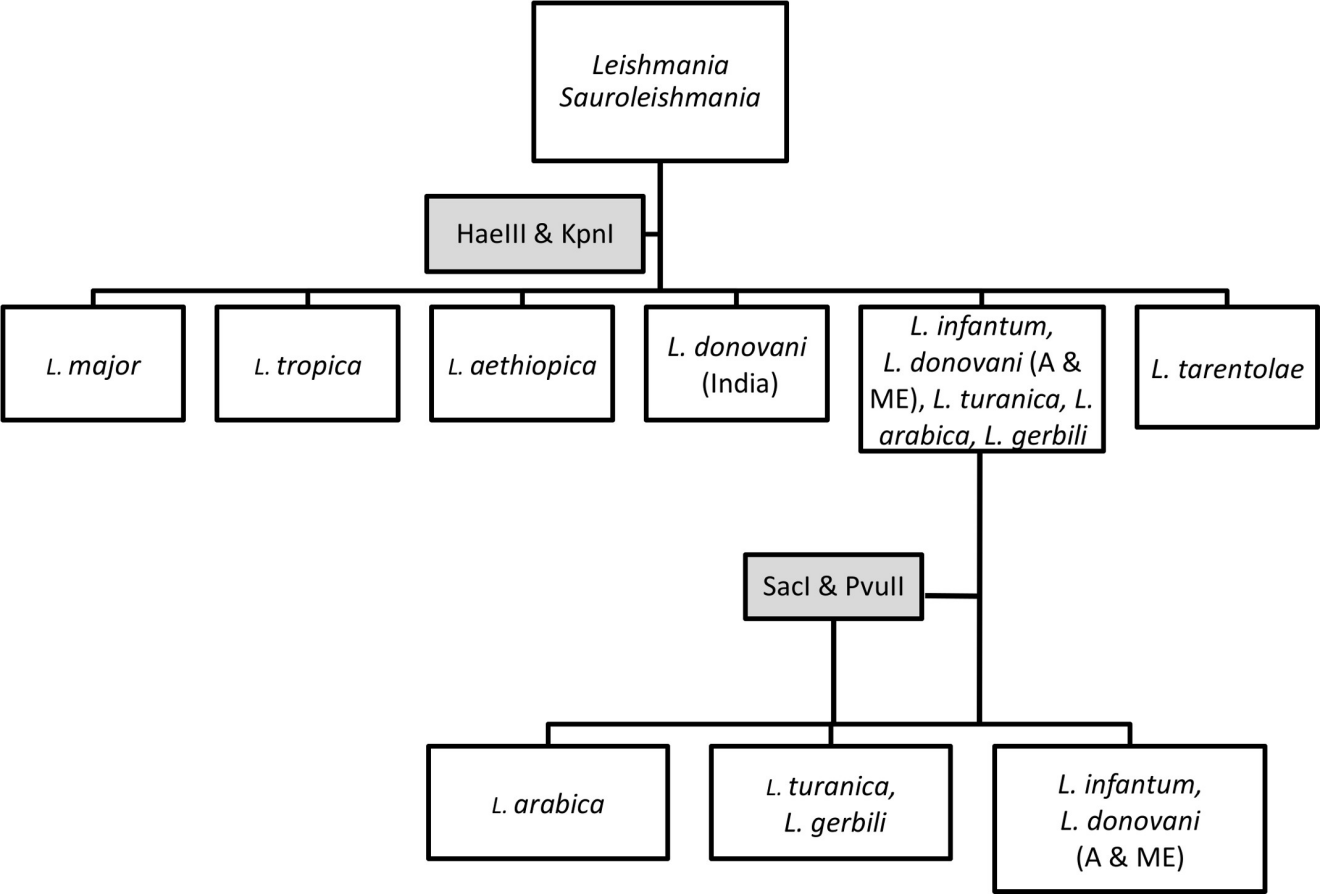
PCR-RFLP algorithm for identification of Old World *Leishmania* species using *DPPIII* gene as target and *DPPIII* F/R primers pair. A: African, ME: Middle Eastern.

In parallel, we took profit of the variable restriction sites to develop PCR-RFLP based identification as an alternative to the sequence analysis. Indeed, to develop the species identification scheme, we used published sequences of Old World *Leishmania* species (*L*. *major*, *L*. *tropica*, *L*. *aethiopica*, *L*. *infantum*, *L*. *donovani*, *L*. *arabica* and *L*. *turanica*) and *L*. (*Sauroleishmania) tarentolae* to identify within the 662bp fragment, the variable restriction sites, which are specific to species- or group of species. We selected four restriction enzymes (RE) (*Hae*III, *Kpn*I, *Pvu*II and *Sac*I) that distinguish between the different *Leishmania* species/group of species according to predicted double- digestion restriction fragments length polymorphisms, as detailed in Table 1 and visualized using the SnapGene 2.8.3 program on S6 Fig. *Leishmania* species identification is possible using the scheme on Fig 5 where up to 2 steps could be taken to identify the *Leishmania* species.

**Table 1:** *In silico* predicted restriction fragments of the *DPPIII* F/R PCR products of the TritrypDB database sequences.

	Endonucleases			
	*Hae*III&*Kpn*I		*Sac*I&*Pvu*II	
*Leishmania* species	Fragments length (bp)	RFLP Pattern	Fragments length(bp)	RFLP Pattern
*L*. *major*	247-193-138-61	P1	486–176	P’1
*L*. *tropica*	247-157-154-42*-39*-22*	P2	486–176	P’1
*L*. *aethiopica*	239-193-157-42*-23*-8*	P3	662-486-176	P’2
*L*. *infantum*	247-199-193-23*	P4	662	P’3
*L*. *donovani*(IN)	440-199-23*	P5	662	P’3
*L*. *turanica*	247-199-193-23*	P4	486–176	P’1
*L*. *arabica*	247-199-193-23*	P4	281-205-176	P’4
*L*. *tarentolae*	463-138-61	P6	486–176	P’1
*L*. *gerbilli*	247-199-193-23*	P4	486–176	P’1

A: African stains, IN: Indian strain; ME: Middle Eastern strains

*Fragments that are not seen on 3% agarose gels

In the first step, the double digestion with *Hae*III and *Kpn*I RE would identify *L*. *major*, *L*. *tropica*, *L*. *aethiopica*, *L*. *tarentolae*, *L*. *donovani* but would not distinguish within the group made of *L*. *infantum*, *L*. *turanica* and *L*. *arabica* (S5A Fig). In a second step, the unresolved DNAs would be digested with *Sac*I and *Pvu*II to differentiate and identify *L*. *infantum*, *L*. *turanica* and *L*. *arabica* (S5B Fig). *L*. *turanica* and *L*. *gerbilli* share the same RFLP patterns.

**Fig 6:**
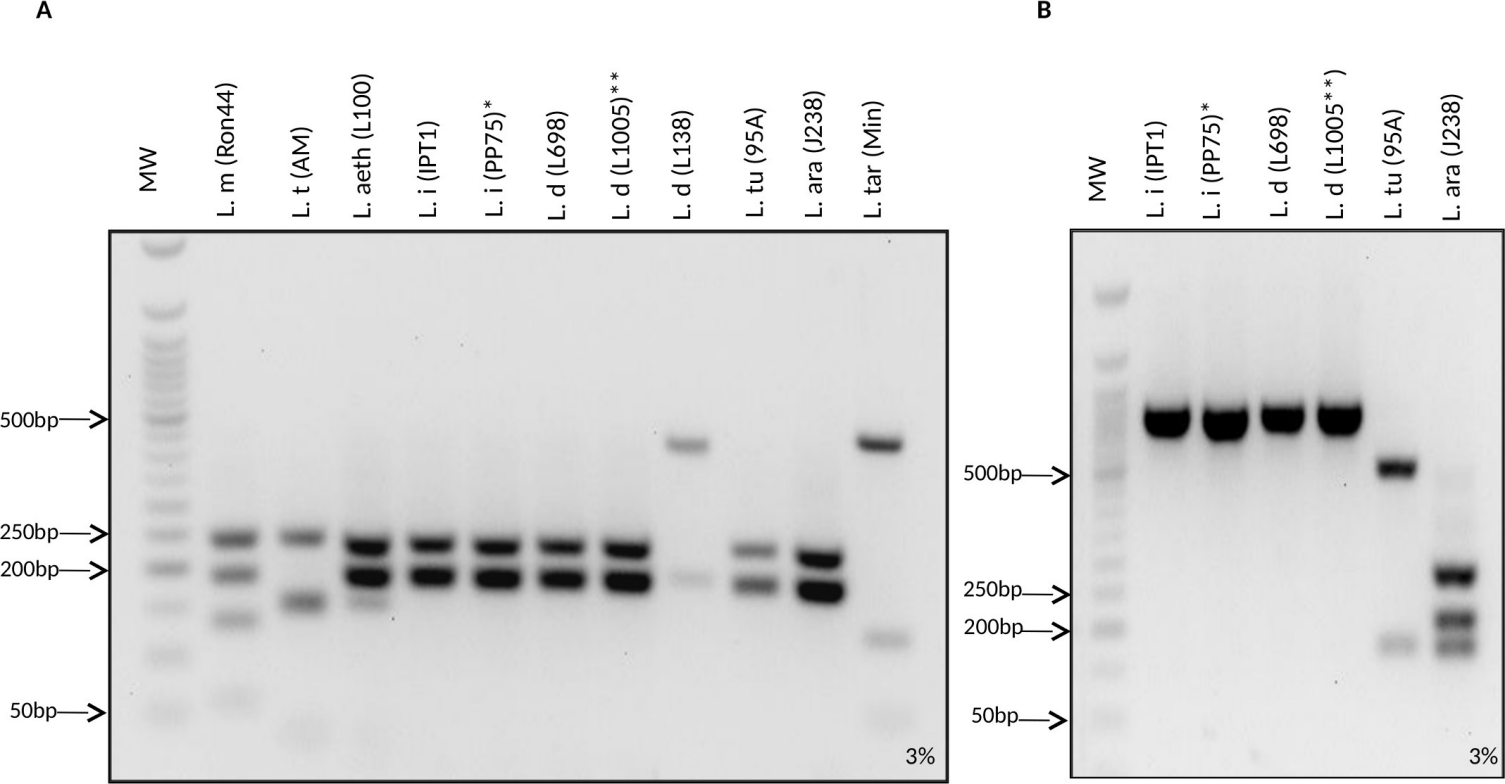
(**A) RFLP patterns of the *Hae*III/*Kpn*I double digested, amplified *DPPIII* fragments.** *L*. *major*, *L*. *tropica*, *L*. *aethiopica*, *L*. *tarentolae*, and Indian *L*. *donovani* presented distinctive profiles while African and Middle Eastern *L*. *donovani*, *L*. *infantum*, *L*. *turanica and L*. *arabica* shared the same patterns. **(B) RFLP patterns of the *Pvu*II/*Sac*I double digested, amplified *DPPIII* fragments.** The *L*. *infantum*, *L*. *arabica* and *L*. *turanica* DNAs were distinguished by their PCR- RFLP profiles. The African and Middle Eastern *L*. *donovani* and *L*. *infantum* DNAs presented the same patterns. MW: 50bp Molecular Weight. *L*. *m*: *L*. *major; L*. *t*: *L*. *tropica; L*.*ae*: *L*. *aethiopica; L*. *i*: *L*. infantum; *Brazilian L. *infantum* strain (also known as *L*. *chagasi*)*; L*. *d*: *L*. donovani; *East African L. *donovani* strain (also known as *L*. *archibaldi*) *; L*. *tu*: *L*. *turanica; L*. *ar*: *L*. *arabica; L*. *tar*: *L*. *tarentolae*.

We validated experimentally this scheme on the selection of DNAs presented on S1 Table corresponding to well- characterized *Leishmania* strains (Table 2). The analysis showed that as expected the double *Hae*III and *Kpn*I digestion allowed the differentiation between *L*. *major*, *L*. *tropica* and *L*. *aethiopica* and their identification while *L*. *infantum*, *L*. *arabica* and *L*. *turanica* species showed the same restriction pattern. Unexpectedly, this pattern was also shared with the African and Middle Eastern *L*. *donovani* strains, and as predicted by the *in silico* analysis, the unique strain tested from India, MHOM/IN/00/DEVI, had a distinctive RFLP pattern (Figs 6A and S5A). This is in accordance with the sequence analyses of the corresponding fragments. The unique *L*. *tarentolae* tested DNA showed a unique profile, different from species of the *Leishmania* subgenus (Fig 6A). Except for the case of *L*. *donovani*, for each species, all tested strains had consistently the same RFLP pattern whatever were their geographical origin, their host or their isolation date, which allows considering these patterns as species-specific (Table 2). The *L*. *infantum*, *L*. *donovani* (African and Middle Eastern), *L*. *arabica* and *L*. *turanica* species DNA products were used in a *Pvu*II and *Sac*I double digestion. We obtained a different and unique restriction pattern for each of *L*. *arabica*, *L*. *turanica* and *L*. *infantum* species DNA as predicted by the *in silico* analysis; the African and Middle Eastern *L*. *donovani* and *L*. *infantum* DNAs shared the same profile (Fig 6B).

**Table 2:** PCR-RFLP patterns of the studied strains.

Species	Code	WHO code	PCR *DPPIII*F/R	*Hae*III-*Kpn*I Patterns	*Sac*I-*Pvu*II Patterns
*L*. *major*	Lipa100	MHOM/DZ/09/LIPA100	+	P1	NA
	SDTBM	ISAL/IN/73/STDBM	+	P1	NA
	GTBM	MMER/IN/73/GTBM	+	P1	NA
	L3171	MHOM/IL/80/Friedlin	+	P1	NA
	IL24	MHOM/IL/83/IL24	+	P1	NA
	IL53	MHOM/IL/83/IL53	+	P1	NA
	KFUH	MHOM/SA/84/KFUH-7532	+	P1	NA
	Ron99	MPSA/TN/87/Ron99	+	P1	NA
	Ron155*	MPSA/TN/ 87/Ron155	+	P1	NA
	FMH	MHOM/TN/90/FMH	+	P1	NA
*L*. *tropica*	K001*	MHOM/AF/82/K001	+	P2	NA
	Lipa155	MHOM/SR/86/LIPA155	+	P2	NA
	Ackerman	MHOM/ASIA/74/Ackerman	+	P2	NA
	LA28	MHOM/GR/00/LA28	+	P2	NA
	DBKM	MCAN/IN/71/DBKM	+	P2	NA
	Bag9	MHOM/IQ/76/BAG9	+	P2	NA
	Bag17	MHOM/IQ/76/BAG17	+	P2	NA
	L75	MHOM/IQ/65/L75	+	P2	NA
	G159	MHOM/IL/00/Gabaï159	+	P2	NA
	Rachnan	MHOM/IL/78/Rachnan	+	P2	NA
	A sinaï III	MHOM/IQ/73/A Sinaï III	+	P2	NA
	AM	MHOM/TN/06/AM	+	P2	NA
	CJ	MHOM/TN/06/CJ	+	P2	NA
	Leep0920	MHOM/TN/09/Leep0920	+	P2	NA
*L*. *aethiopica*	L100	MHOM/ET/72/L100	+	P3	P’2
*L*. *infantum*	Lipa1153*	MHOM/DZ/01/LIPA1153	+	P4	P’3
	Lipa506	MHOM/DZ/96/LIPA506	+	P4	P’3
	Lipa222	-	+	P4	P’3
	MW111	MHOM/SD/00/MW111	+	P4	P’3
	LLM805	MCAN/ES/98/LLM805	+	P4	P’3
	LLM662	MCAN/ES/97/LLM662	+	P4	P’3
	IPT1	MHOM/TN/80/IPT1	+	P4	P’3
	LV50*	MHOM/TN/94/LV50	+	P4	P’3
	LV49	MHOM/TN/94/LV49	+	P4	P’3
	Drep14	MHOM/TN/97/Drep14	+	P4	P’3
	Drep05	MHOM/TN/96/Drep05	+	P4	P’3
	IPT1	MHOM/TN/80/IPT1	+	P4	P’3
	KA413	MHOM/TN/87/KA 413	+	P4	P’3
	KA439	MHOM/TN/88/KA 439	+	P4	P’3
	Drep11	MHOM/TN/97/Drep11	+	P4	P’3
	Drep13	MHOM/TN/97/Drep13	+	P4	P’3
	Drep08	MHOM/TN/96/Drep08	+	P4	P’3
	Drep15	MHOM/TN/98/Drep15	+	P4	P’3
	LVGA	MHOM/TN/95/LVGA	+	P4	P’3
	PP75	MHOM/BR/1974/PP75	+	P4	P’3
*L*. *donovani*	LEM138	MHOM/IN/00/DEVI	+	P5	NA
	LEM698	MHOM/ET/67/HU3	+	P4	P’3
	LEM496	MHOM/KE/75/H9	+	P4	P’3
	LEM719	IMRT/KE/62/LRC-L57	+	P4	P’3
	LEM536	MHOM/SA/81/JEDDAH-KA	+	P4	P’3
	MW29	MHOM/SD/00/MW29	+	P4	P’3
	L1005	MHOM/ET/72/GEBRE1	+	P4	P’3
	MW09	MHOM/SD/00/MW09	+	P4	P’3
	MW23	MHOM/SD/00/MW23	+	P4	P’3
	MW26	MHOM/SD/00/MW26	+	P4	P’3
	MW81	MHOM/SD/00/MW81	+	P4	P’3
*L*. *turanica*	95A	MRHO/SU/74/95A	+	P4	P’1
*L*. *arabica*	Jisha238	MPSA/SA/84/Jisha238	+	P4	P’4
*L*. *tarentolae*	Min I	IMIN/IT/86/MIN1	+	P6	NA

NA: Not Analyzed

### PCR-RFLP identification of parasites within suspected human cutaneous leishmaniasis samples

**Fig 7:**
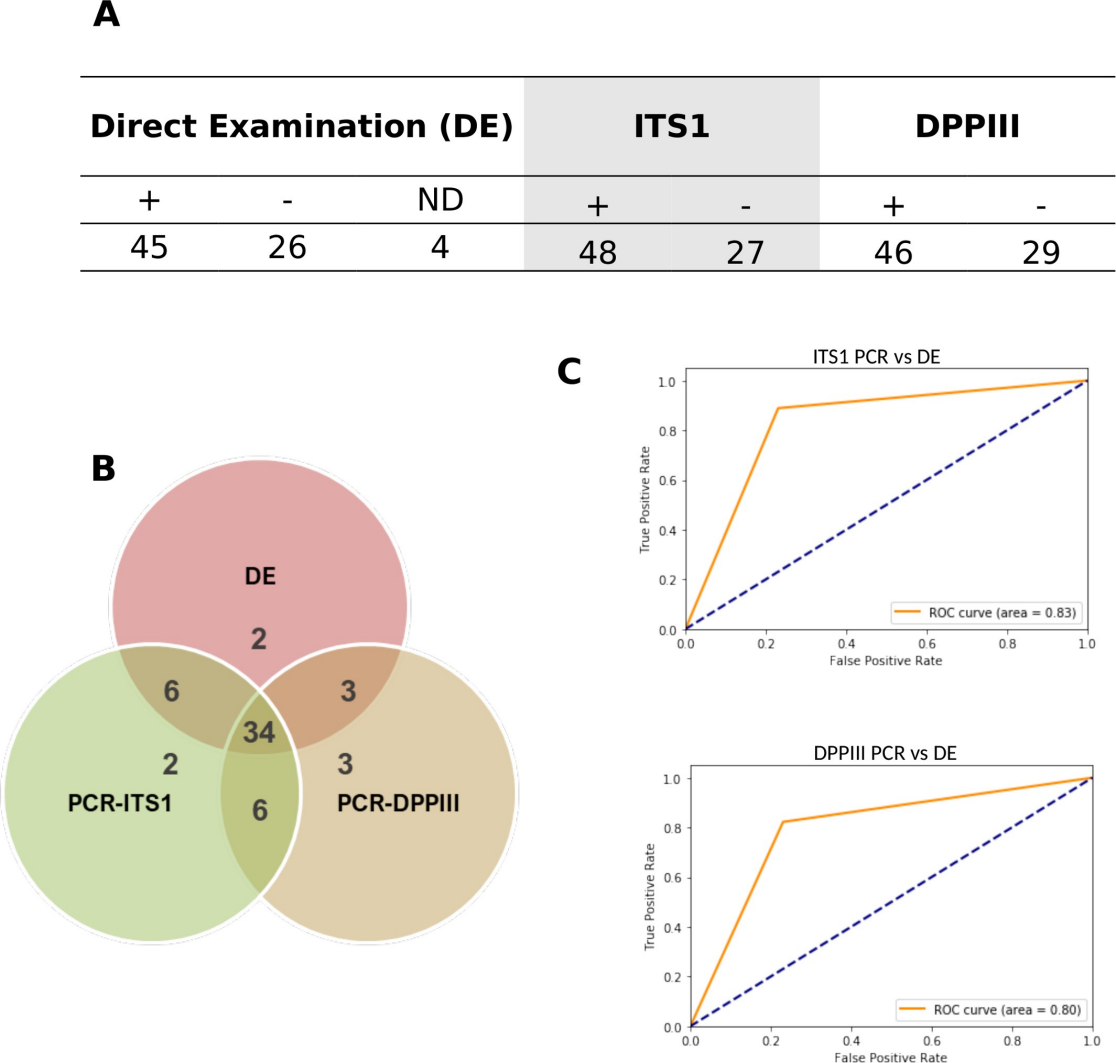
**(A)** Results of CL samples. **(B)**Venn Diagram showing concordance of detected *Leishmania* parasites on 71 CL samples between DE, PCR-ITS1 and PCR-DPPIII. **(C)** Performances measuring of DPPIII and ITS1 PCRs versus Direct examination using ROC curves. ND: Not determined.

To validate use of our *DPPIII* PCR and RFLP assays for *Leishmania* parasites detection and identification in clinical samples, we tested 75 DNAs of cutaneous samples taken from patients referred to clinicians for CL diagnosis during the 2010–2018 period. We also used ITS1 PCR-RFLP on the same sample and compared the results to direct smear examination (DE) as gold standard. The S2 Table gathers all the results obtained with these samples. As shown on Fig 7A, out of the 75 samples, 48 were ITS1 PCR^**+**^ and 46 *DPPIII* PCR^**+**^. The distribution of positive results across the 3 tests on all the samples is shown on Fig 7B. Six samples that were ITS1 PCR^**-**^ were *DPPIII* PCR^**+**^. However, 8 ITS1 PCR^**+**^ samples were *DPPIII* PCR^**-**^. Two DE^**+**^ samples were negative by both PCR assays. Measured sensitivity using DE as gold standard was 82.22% for DPPIII and 88.8% for ITS1. Specificity was estimated to be 76.92% for both methods. Computed areas under the curve (AUC_ROC) were 0.80 and 0.83 respectively (Fig 7C).

The RFLP analysis of the amplicons identified 39 samples out of the 46 DPPIII positives and 47 among the 48 ITS1 positives. S7 Fig illustrates some of the identification results made by the *DPPIII* PCR *Hae*III/*Kpn*I RFLP. The two molecular identification methods had 100% agreement as regards *Leishmania* species identification (S2 Table).

## Discussion

There is need for novel molecular tools to respond to challenges associated to leishmaniases and continuous changes in their epidemiology at local and global levels [12,20,21,66]. Indeed, molecular tools are instrumental to define strategies for disease control as they allow identification and characterization of transmitted strains or species and use of genetic diversity for spatial and temporal tracking and comparison of strains. The use of DNA markers is the alternative of choice to laborious, costly and time consuming MLEE methods [25,67,68]. During the last decade, this technique was increasingly replaced by other efficient molecular approaches such as MLST [69–72] and MLMT [40–42] and whole genome sequences [73,74] to study genetic diversity, epidemiology, population genetics and evolution of *Leishmania* parasites. However, these methods remain laborious and expensive. So, referring to individual markers for *Leishmania* molecular characterization, epidemiology and phylogenetic studies remains an alternative of choice despite the lower resolution for population genetic studies. When single markers evolution reflects *Leishmania* parasites phylogeny, their sequence analysis or use are effective in a congruent species assignment to MLST or MLEE identification [25,39,75].

Dipeptidyl Peptidase III protein coding sequence has been identified as a potential target for DNA parasite identification. The gene is present in the genome of *Leishmania*, *Endotrypanum*, *Crithidia*, *Leptomonas* and *Blechomonas* [50] but is lost in the *Trypanosoma* genus including *T*. *brucei* and *T*. *cruzi* human pathogens [52]. Interestingly, although the DNA sequence is present in the *L*. *(Sauroleishmania)* subgenus represented by *L*. *tarentolae* species and annotated as a putative *DPPIII* protein, the CDS is initiated much downstream from the ATG site in other *Leishmania* genes, resulting in a shorter coding region (470bp vs 2040bp).

We previously used *DPPIII* gene as a target to amplify a 664bp fragment from *L*. *major*, *L*. *tropica*, *L*. *arabica and L*. *aethiopica*, while the PCR was negative for strains of *L*. *infantum*, *L*. *donovani*, and *L*. *tarentolae* species [53]. Thus, the main objective of this study was to further characterize and validate the *DPPIII* gene as a molecular marker for Old World *Leishmania* taxonomy and molecular epidemiology. For this purpose, we used sequences or strains belonging to a range of pathogenic, non- pathogenic (e.g. *L*. *turanica*, *L*. *arabica*, *L*. *gerbilli*, *L*. *tarentolae*) *Leishmania* species to humans, and monoxenous kinetoplastids (*Leptomonas pyrrocoris*, *Leptomonas seymouri)* that also could be opportunistic to *Leishmania* species in humans [76–78]. We attempted to cover different *Leishmania* taxa, geographical origins, hosts and diseases resulting in a representative set of DNAs of pathogenic *Leishmania* species in the OW; *L*. *aethiopica* and non-pathogenic *Leishmania* were represented each by a unique DNA.

*Leishmania* genus evolution is mainly marked by the development of a digenetic life cycle and the introduction of the intracellular amastigote stage in the development cycle [79]. In this study, the phylogenetic analysis of the entire gene has shown a congruent grouping of the parasites to the genus and subgenus level [79], or *Leishmania* MLSA groups [80] and the reported species assignment of the parasites. The sequence analysis of the 662bp fragment of the *DPPIII* gene that contains the zinc binding motifs of the active site, in 69 *Leishmania* DNAs and 19 Database sequences, also showed a clear separation between the New World (*L*. *mexicana*, *L*. *braziliensis*, *L*. *panamensis* and *L*.*enriettii*) and the Old World species (*L*. *major*, *L*. *tropica*, *L*. *aethiopica*, *L*. *turanica*, *L*. *gerbilli*, *L*. *arabica* and *L*. *infantum/ L*. *donovani*). *L*. *tarentolae* was grouped with Old World species which is in accordance with the most recent classification [10].

Interestingly, the analysis also suggested that the gene is under negative selection, a known feature of essential genes [81] that corroborates the physiological importance of the protein [50]. This feature, also observed for the 662bp sequenced fragments, thus infers a potential stability of the observed haplotypes. Among the twenty haplotypes observed, some species had more than one sequence type, some mutations seemed to be species specific and may constitute potential bioinformatics species signatures.

The two sequence types that were observed for *L*. *major* were different from those of the closely related, supposedly non- pathogenic to human, *L*. *arabica*, *L*. *turanica* and *L*. *gerbilli*. These species, known to be endemic in Middle East and Central Asia, share the same transmission areas and even cycle components as *L*. *major*; mixed infections were even described [82–86]. The sequences of these 3 species were also different from one another.

Two sequences types were also observed for *L*. *infantum*, and *L*. *donovani* which is a closely related species. These parasites have a still debated taxonomy. While some studies described them as two distinct groups [87,88], others considered that they should be considered as one species complex (*L*. *donovani* complex) due to the low genetic diversity between them [43,47]. In addition, in a genome-wide global study that included Indian, East African and Middle Eastern strains, the phylogenetic reconstruction of the *L*. *donovani* complex based on SNP variations clearly separated these parasites into five major groups that coincided with their geographic origin [74]. Microsatellites and enzyme coding genes analyses have also described the Indian strains of *L*. *donovani* as a distinct genetic sub-group from East African strains [89,90]. In another study based on *cpB* sequence analysis, Sudanese and Ethiopian *L*. *donovani* strains constituted a distinct subgroup from other *L*. *donovani* complex strains that included *L*. *infantum* from Tunisia and *L*. *donovani* from Kenya, India, and Iran [33]. In this study, two specific SNPs differentiated the Indian *L*. *donovani* strains from the North (*L*. *infantum*) and East African (*L*. *donovani*) ones, corroborating close relatedness of the African parasites of this species complex, not allowing to identify an *L*. *infantum* sequence type.

The *DPPIII* fragment sequence analyses also showed that, among the Old World studied species, *L*. *tropica* was the most polymorphic. Indeed, we noticed the presence of 5 haplotypes corresponding to 3 different geographical origins. The first haplotype was observed for the Mediterranean strains from Tunisia and Greece; three other haplotypes corresponded to Middle Eastern parasites, and the last one was shared between the unique Indian strain and a Middle Eastern one. Geographical subdivision within *L*. *tropica* was also observed in other studies using more sophisticated methods such as microsatellites [91] and genome sequence analyses [92].

We tested only one DNA in case of *L*. *aethiopica* and non- pathogenic *Leishmania* species. Our findings would need to be validated using additional strains. However, we have observed that each of their sequences were sufficiently different from the other studied ones to be distinguished. *L*. (*Sauroleishmania*), including *Leishmania tarentolae* species, are reptile parasites, described in different evolution studies as deriving from the mammalian forms of *Leishmania* [89,90,93], and recently recognized as a subgenus of the *Leishmania* genus [10]. *L*. *tarentolae* was detected and identified in hard and soft tissues of a 300 years old Brazilian mummy, emphasizing the fact that these parasites might infect and visceralize in humans [94]. This species is often overlooked in epidemiological investigations and little work describes the impact of its presence in transmission foci. Ability to detect and identify non-pathogenic species is cornerstone to gain insights into established Leishmaniases transmission cycles or in epidemiological investigations.

In this study, we further validated DPPIII gene as a *Leishmania* taxonomy marker by developing a new *DPPIII* PCR-RFLP based parasite identification where one or two steps would be needed for taxonomical assignments of the parasites in the Old World. Thereby, *Hae*III/*Kpn*I double digestion allows successful identification of *L*. *major*, *L*. *tropica*, *L*. *aethiopica*, the group representing *L*. *infantum* and *L*. *donovani* species, and even the species *L*. *tarentolae* of the subgenus *L*. (*Sauroleishmania)*. Depending on the studied geographical region, this first analysis step could be sufficient to identify the endemic species making the assays cheaper and quicker. However, exceptions will be if imported species are observed and studied, or, in case of Asian and Middle Eastern regions, where *L*. *turanica* and *L*. *arabica* are respectively co-sympatric to *L*. *major*, and co-endemic with *L*. *infantum* or *L*. *donovani*. A second *Sac*I/*Pvu*II digestion step will precise the species identity that could not be ascertained during the first step. Although the *L*. *gerbilli* and *L*. *turanica* sequences are different, the selected enzymes did not generate discriminating RFLP patterns. Therefore, for these parasite species the sequence analysis is more appropriate for their identification.

In previous studies, PCR-RFLP analysis using a single marker like gp63 [27], cytB [95], and hsp70 coding sequences [26] could not differentiate between *L*. *tropica* and *L*. *aethiopica* species. However it was possible to differentiate them using species- specific PCR and MLMT assays [96,97]. In another study, discrimination of these two species required the concomitant analysis of two markers, hsp20 and hsp70 sequences [25]. Here, we were able to distinguish between the two species by a double digestion (*Hae*III & *Kpn*I) of a single maker, and we recommend the validation of its use for the identification of *L*. *aethiopica*. Using ITS1 PCR-*Hae*III RFLP method [55], *L*. *aethiopica* digestion profile looked remarkably like the *L*. *infantum/L*.*donovani* species profile. It needed the use of metaphor agarose gel electrophoresis or a second digestion step (using *CfoI*) to differentiate these species. In case of DPPIII-RFLP assay, we recommend use of 2% - 3% agarose gel electrophoresis to achieve optimal resolution of the *L*. *aethiopica* RFLP pattern so it can be clearly distinguished from the *L*. *donovani* one (S8 Fig).

In addition, as expected from the sequence analyses, *L*. *infantum* and East African *L*. *donovani* strains shared the same RFLP pattern. Interestingly, RFLP of single markers like *HSP70* did not differentiate these two species [25,98]. However, using PCR-RFLP ITS1-*Hae*III, a slight size difference was observed in RFLP patterns between *L*. *infantum* and *L*. *donovani* when a prolonged 2% metaphor agarose gel electrophoresis was performed [55]. Differences in RFLP patterns between the two species was also observed when intragenic *gp63* PCR was followed by an *Msc*I restriction, and when *Sal*I was used the 3 species *L*. *infantum*, *L*. *donovani* and *L*. *archibaldi* (Sudanese and Ethiopian *L*. *donovani*) presented distinct profiles. But Southern blotting and radioactive hybridization were required to allow full discriminative resolution of the digestion profiles, notably to detect the small sized bands that had faint intensity. An alternative for detection of these bands was the use of specific conditions for the electrophoresis (time, %, agarose brand) [27].

The application of *DPPIII* PCR on clinical samples demonstrated its ability to detect *Leishmania* parasites in DE^**+**^ human samples without reacting with human DNA as shown in *Leishmania* negative samples. The performances of this method were shown close to those of ITS1 PCR using direct examination diagnosis as gold standard, although sensitivity of ITS1 was higher. Their AUC ROC also pointed to the ability of these tests to distinguish True Positive from False Positive cases. The performance difference could be due to copy number of the targets, here a single copy *DPPIII* gene versus multi-copy ITS1, or also by the fact that our target sequence (662bp) is longer than the ITS1 one (300-350bp). In a similar context, reducing the target length was shown to greatly improve the sensitivity of PCR assays [98]. Interestingly, the *DPPIII* PCR was able to detect 3 DE^**+**^ samples that were not detected by ITS1 PCR despite the repeated nature of this target. Multicopy or repetitive sequences, notably intergenic and spacer regions, could be prone to sequence variations and more rapid accumulation of mutations than in coding regions [99]. Interestingly, hypothesis of occurrence of mutations in priming sites of ITS2 sequences was proposed to explain lack of amplification in infected samples [100]. Heterogeneity of ITS1 sequences was the cause of unreadable sequences [101]. RFLP patterns of multicopy sequences (e.g.*Cpb*) could also be difficult to analyze due to complexity created by isogene variations [39]. In case of the samples here tested, RFLP profiles obtained with both *DPPIII* and ITS1 amplified products were relatively easy to analyze, although in other ongoing studies it was not uncommon for us to observe difficult to interpret ITS1 RFLP profiles hampering *Leishmania* identification. We could identify the *Leishmania* species using one RFLP step in case of 39 *DPPIII*^**+**^ samples, while in case of 7 the amplicons were in too low amount to be further processed.

In the present study, we demonstrated by a phylogenetic approach that the *DPPIII* gene is a suitable marker for taxonomy and demonstrated ability of the developed *DPPIII* PCR to detect and identify parasites within human samples. So it could be used for diagnosis and molecular epidemiology studies to complement other methods including the ITS1 PCR assay. Indeed, it is recommended to use a combination of markers and techniques to provide an accurate diagnosis [102] and to achieve the aimed taxonomy resolution [10,102] in case of migration, travel or emergence.

## Conclusion

Application of molecular identification methods for robust *Leishmania* taxonomical identification constitutes a key step to conduct molecular epidemiology investigations, to monitor disease outbreak and spread, and to implement rapid and adequate preventive measures. Molecular analyses of the *DPPIII* gene demonstrate it to be a plausible complement / alternative for such purposes in North Africa. Further investigations need be done to assess gene diversity in the under-represented species of this study, especially *L*. *donovani* in the Indian subcontinent, *L*. *aethiopica* in Africa, and *L*. *turanica*, *L*. *gerbilli* and *L*. *arabica* in Asian and Middle Eastern foci. This marker may contribute to advance in the fight against leishmaniases.

### Data bases

TritrypDB (https://tritrypdb.org/tritrypdb/)
The Restriction Enzyme Database (REBASE) **(**http://rebase.neb.com/)

### Softwares

Geneious v3.6.2 (www.geneious.com)
NetPrimer (http://www.premierbiosoft.com/netprimer/)
MEGA 6 (https://www.megasoftware.net/)
SnapGene (www.snapgene.com)
DNA Baser sequence assembler v4 program (www.DnaBaser.com)

## Supporting information

S1 Fig Generic amplification of a 662bp DPPIII fragment from the studied *Leishmania* species. MW: 100bp Molecular Weight. *L*.*m*: *L*. *major; L*.*t*: *L*. *tropica; L*.*ae*: *L*. *aethiopica; L*.*i*: *L*. infantum; *Brazilian *L*. *infantum* strain(also known *as L*. *chagasi*)*; L*.*d*: *L*. donovani; **East African L. *donovani strain (also known as L*. *archibaldi); L*.*tu*: *L*. *turanica; L*.*ar*: *L*. *arabica; L*.*tar*: *L*. *tarentolae*.
(TIF)

S2 Fig Evolutionary analysis of *Leishmania* parasites using the Maximum Likelihood method. The evolutionary history of *Leishmania* parasites was inferred by using the Maximum Likelihood method and Tamura 3-parameter model. The bootstrap consensus tree inferred from 1000 replicates is taken to represent the evolutionary history of the taxa analyzed. Branches corresponding to partitions reproduced in less than 50% bootstrap replicates are collapsed. The percentage of replicate trees in which the associated taxa clustered together in the bootstrap test (1000 replicates) are shown next to the branches. Initial tree(s) for the heuristic search were obtained automatically by applying Neighbor-Join and BioNJ algorithms to a matrix of pairwise distances estimated using the Tamura 3 parameter model, and then selecting the topology with superior log likelihood value. A discrete Gamma distribution was used to model evolutionary rate differences among sites (5 categories (+*G*, parameter = 0.3096)). This analysis involved 72 nucleotide sequences. Codon positions included were 1st+2nd+3rd+Noncoding. There were a total of 662 positions in the final dataset. Evolutionary analyses were conducted in MEGA X.
(TIF)

S3 Fig Evolutionary relationships of *Leishmania* parasites using the Neighbor-Joining method. The bootstrap consensus tree inferred from 1000 replicates is taken to represent the evolutionary history of the taxa analyzed. Branches corresponding to partitions reproduced in less than 50% bootstrap replicates are collapsed. The percentage of replicate trees in which the associated taxa clustered together in the bootstrap test (1000 replicates) are shown next to the branches. The evolutionary distances were computed using the Tamura 3-parameter method and are in the units of the number of base substitutions per site. The rate variation among sites was modeled with a gamma distribution (shape parameter = 0.65). This analysis involved 72 nucleotide sequences. Codon positions included were 1st+2nd+3rd+Noncoding. All ambiguous positions were removed for each sequence pair (pairwise deletion option). There were a total of 662 positions in the final dataset. Evolutionary analyses were conducted in MEGA X.
(TIF)

S4 Fig Evolutionary relationships of *Leishmania* parasites using the Minimum Evolution method. The bootstrap consensus tree inferred from 1000 replicates is taken to represent the evolutionary history of the taxa analyzed. Branches corresponding to partitions reproduced in less than 50% bootstrap replicates are collapsed. The percentage of replicate trees in which the associated taxa clustered together in the bootstrap test (1000 replicates) are shown next to the branches. The evolutionary distances were computed using the Tamura 3-parameter method and are in the units of the number of base substitutions per site. The rate variation among sites was modeled with a gamma distribution (shape parameter = 0.65). The ME tree was searched using the Close-Neighbor-Interchange (CNI) algorithm at a search level of 1. The Neighbor-joining algorithm was used to generate the initial tree. This analysis involved 72 nucleotide sequences. Codon positions included were 1st+2nd+3rd+Noncoding. All ambiguous positions were removed for each sequence pair (pairwise deletion option). There were a total of 662 positions in the final dataset. Evolutionary analyses were conducted in MEGA X.
(TIF)

S5 Fig Evolutionary relationships of *Leishmania* parasites using the UPGMA method. The bootstrap consensus tree inferred from 1000 replicates is taken to represent the evolutionary history of the taxa analyzed. Branches corresponding to partitions reproduced in less than 50% bootstrap replicates are collapsed. The percentage of replicate trees in which the associated taxa clustered together in the bootstrap test (1000 replicates) are shown next to the branches. The evolutionary distances were computed using the Tamura 3-parameter method and are in the units of the number of base substitutions per site. The rate variation among sites was modeled with a gamma distribution (shape parameter = 0.65). This analysis involved 72 nucleotide sequences. Codon positions included were 1st+2nd+3rd+Noncoding. All ambiguous positions were removed for each sequence pair (pairwise deletion option). There were a total of 662 positions in the final dataset. Evolutionary analyses were conducted in MEGA X.
(TIF)

S6 Fig *In silico* predicted DPPII PCR-RFLP patterns. A. After double digestion with HaeIII&KpnI endonucleases. B. After double digestion with SacI and PvuII of the PCR products. The parasites that could not be distinguished by HaeIII/KpnI digestion are differentiated by the SacI/PvuII restriction. MW: 100 bp Molecular Weight. *L*.*m*: *L*. *major*, *L*.*i*: *L*. *infantum*, *L*.*t*: *L*. *tropica*, *L*.*d*: *L*. *donovani*, *L*.*ae*: *L*. *aethiopica*, *L*.*ar*: *L*. *arabica*, *L*.*tu*: *L*. *turanica*, *L*.*ta*: *L*. *tarentolae*.
(TIF)

S7 Fig Identification of *Leishmania* species in clinical material using DPPII-PCR and restriction enzyme analysis. DNA was isolated directly from clinical samples, amplified by DPPIII-PCR and double digested with *HaeIII-KpnI*. MW: 50bp Molecular Weight. *L*. *m*: *L*. *major* (IL24), *L*. *i*: *L*. *infantum* (Drep 5) and *L*. *t*: *L*. *tropica* (LA28). Lanes 1–13: Clinical samples.
(TIF)

S8 Fig (A) Second round digestion of L. aethiopica strain. MW: 50bp Molecular weight; L. i: L. infantum (LV08); L. d: L. donovani (L1005); L. tu: L. turanica (95A); L. ara: L. arabica (J238); L. aeth: L. aethiopica (L100). (B) Two round digestion of L. aethiopica strain visualized on a 2% agarose gel. Lane 1: HaeII-KpnI double digestion, Lane 2: SacI-PvuII double digestion.
(TIF)

S1 Table Selection of studied strains. (DOCX)

S2 Table Human CL samples. (XLSX)

S3 Table Selection of 19 gene sequences taken from the public TritrypDB databases and used for the typing scheme elaboration. (DOCX)

S4 Table Variable sites in the *DPPIII* gene between *Leishmania* species. (XLSX)

S5 Table Variable sites in the 662bp fragment and the associated haplotypes of the studied *Leishmania* species. (XLSX)
