# Supplementary figures and images for: Dipeptidyl peptidase III as a DNA marker to investigate epidemiology and taxonomy of Old World Leishmania species
Source: PLoS Negl Trop Dis. 2021 Jul 26;15(7):e0009530. doi: 10.1371/journal.pntd.0009530 (PMC8341715; doi:10.1371/journal.pntd.0009530)

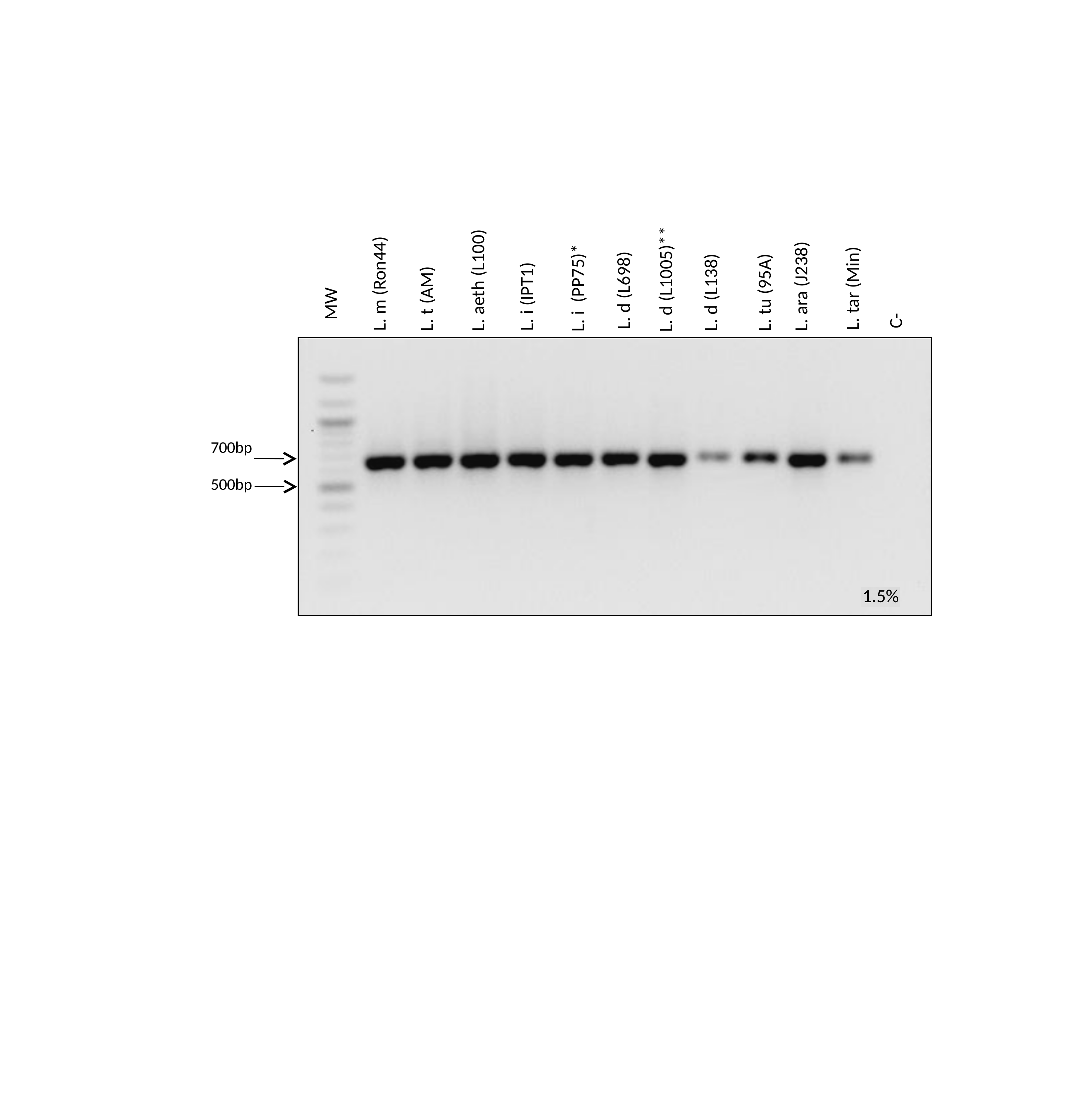

Supplement: S1 Fig — MW: 100bp Molecular Weight. L.m: L. major; L.t: L. tropica; L.ae: L. aethiopica; L.i: L. infantum; *Brazilian L. infantum strain(also known as L. chagasi); L.d: L. donovani; **East African L. donovani strain (also known as L. archibaldi); L.tu: L. turanica; L.ar: L. arabica; L.tar: L. tarentolae. (TIF) [file pntd.0009530.s001.tif]

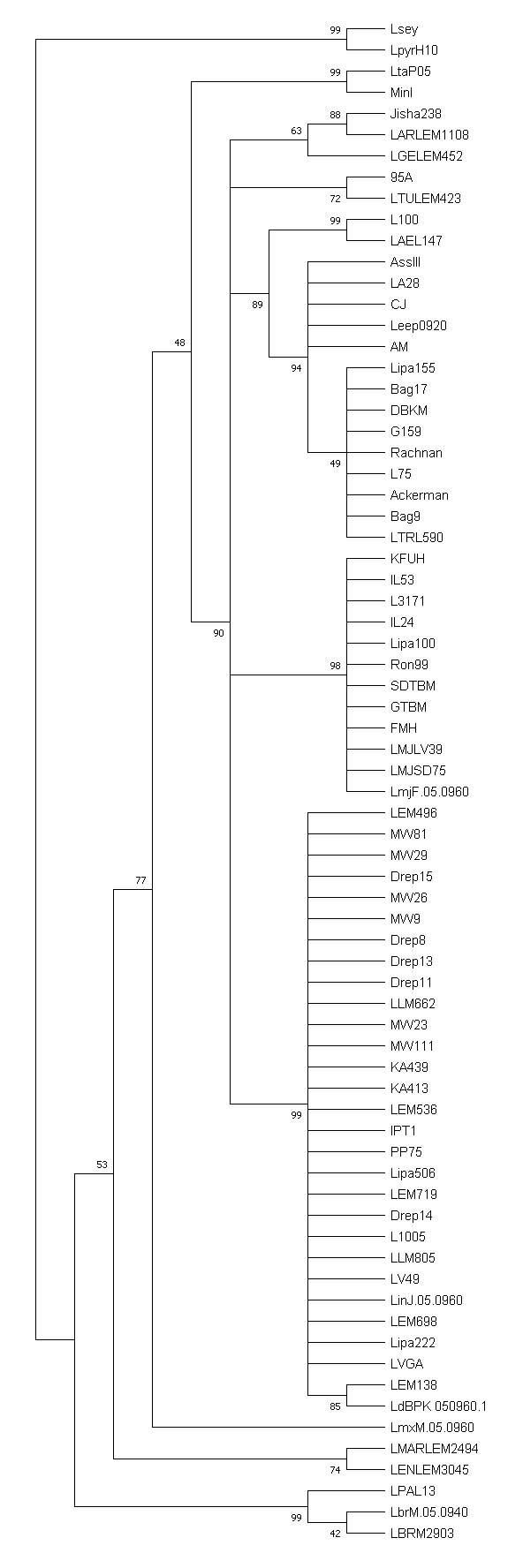

Supplement: S2 Fig — The evolutionary history of Leishmania parasites was inferred by using the Maximum Likelihood method and Tamura 3-parameter model. The bootstrap consensus tree inferred from 1000 replicates is taken to represent the evolutionary history of the taxa analyzed. Branches corresponding to partitions reproduced in less than 50% bootstrap replicates are collapsed. The percentage of replicate trees in which the associated taxa clustered together in the bootstrap test (1000 replicates) are shown next to the branches. Initial tree(s) for the heuristic search were obtained automatically by applying Neighbor-Join and BioNJ algorithms to a matrix of pairwise distances estimated using the Tamura 3 parameter model, and then selecting the topology with superior log likelihood value. A discrete Gamma distribution was used to model evolutionary rate differences among sites (5 categories (+G, parameter = 0.3096)). This analysis involved 72 nucleotide sequences. Codon positions included were 1st+2nd+3rd+Noncoding. There were a total of 662 positions in the final dataset. Evolutionary analyses were conducted in MEGA X. (TIF) [file pntd.0009530.s002.tif]

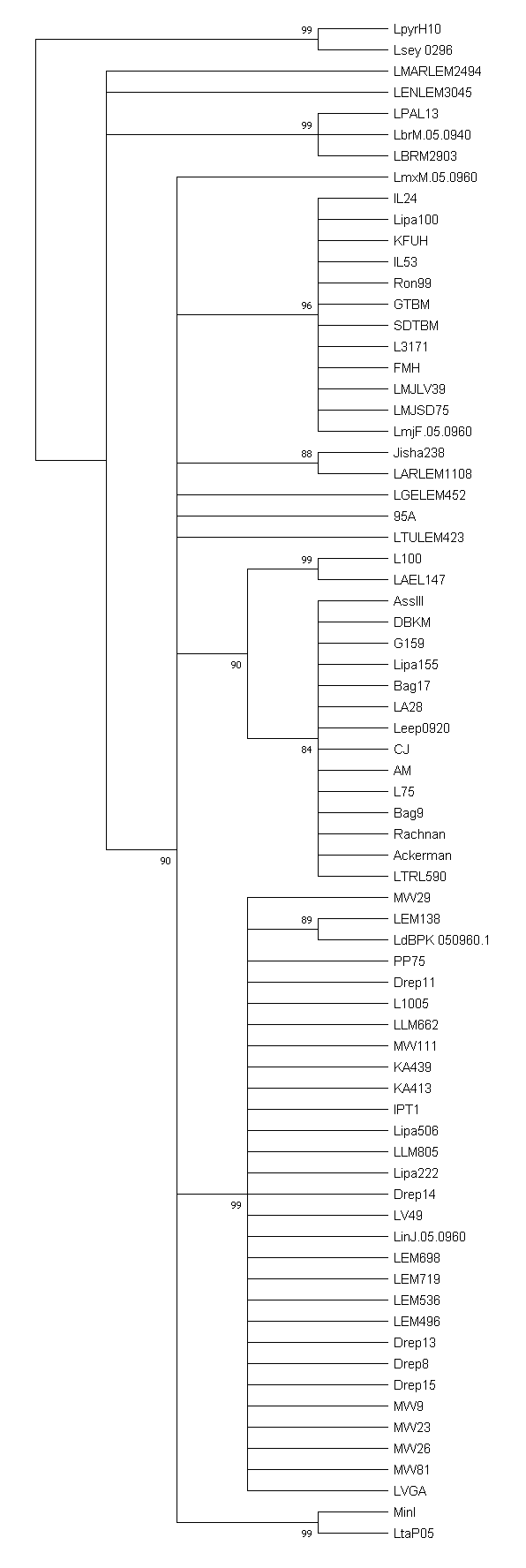

Supplement: S3 Fig — The bootstrap consensus tree inferred from 1000 replicates is taken to represent the evolutionary history of the taxa analyzed. Branches corresponding to partitions reproduced in less than 50% bootstrap replicates are collapsed. The percentage of replicate trees in which the associated taxa clustered together in the bootstrap test (1000 replicates) are shown next to the branches. The evolutionary distances were computed using the Tamura 3-parameter method and are in the units of the number of base substitutions per site. The rate variation among sites was modeled with a gamma distribution (shape parameter = 0.65). This analysis involved 72 nucleotide sequences. Codon positions included were 1st+2nd+3rd+Noncoding. All ambiguous positions were removed for each sequence pair (pairwise deletion option). There were a total of 662 positions in the final dataset. Evolutionary analyses were conducted in MEGA X. (TIF) [file pntd.0009530.s003.tif]

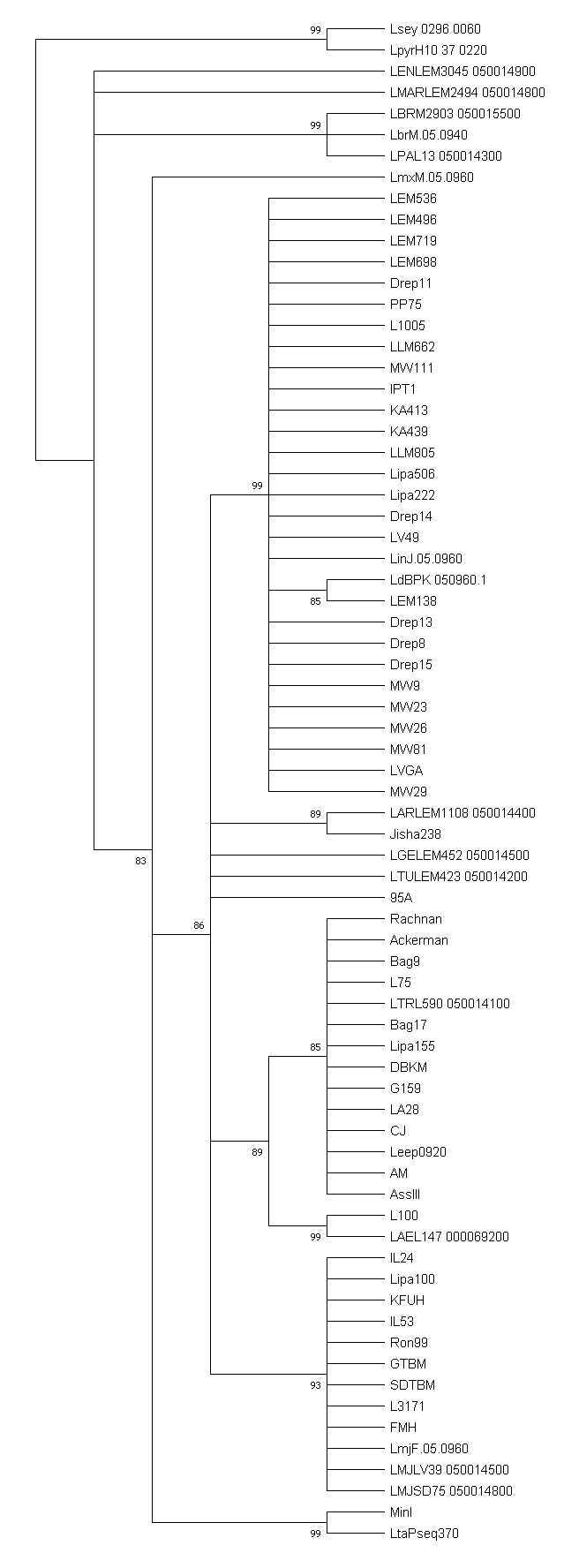

Supplement: S4 Fig — The bootstrap consensus tree inferred from 1000 replicates is taken to represent the evolutionary history of the taxa analyzed. Branches corresponding to partitions reproduced in less than 50% bootstrap replicates are collapsed. The percentage of replicate trees in which the associated taxa clustered together in the bootstrap test (1000 replicates) are shown next to the branches. The evolutionary distances were computed using the Tamura 3-parameter method and are in the units of the number of base substitutions per site. The rate variation among sites was modeled with a gamma distribution (shape parameter = 0.65). The ME tree was searched using the Close-Neighbor-Interchange (CNI) algorithm at a search level of 1. The Neighbor-joining algorithm was used to generate the initial tree. This analysis involved 72 nucleotide sequences. Codon positions included were 1st+2nd+3rd+Noncoding. All ambiguous positions were removed for each sequence pair (pairwise deletion option). There were a total of 662 positions in the final dataset. Evolutionary analyses were conducted in MEGA X. (TIF) [file pntd.0009530.s004.tif]

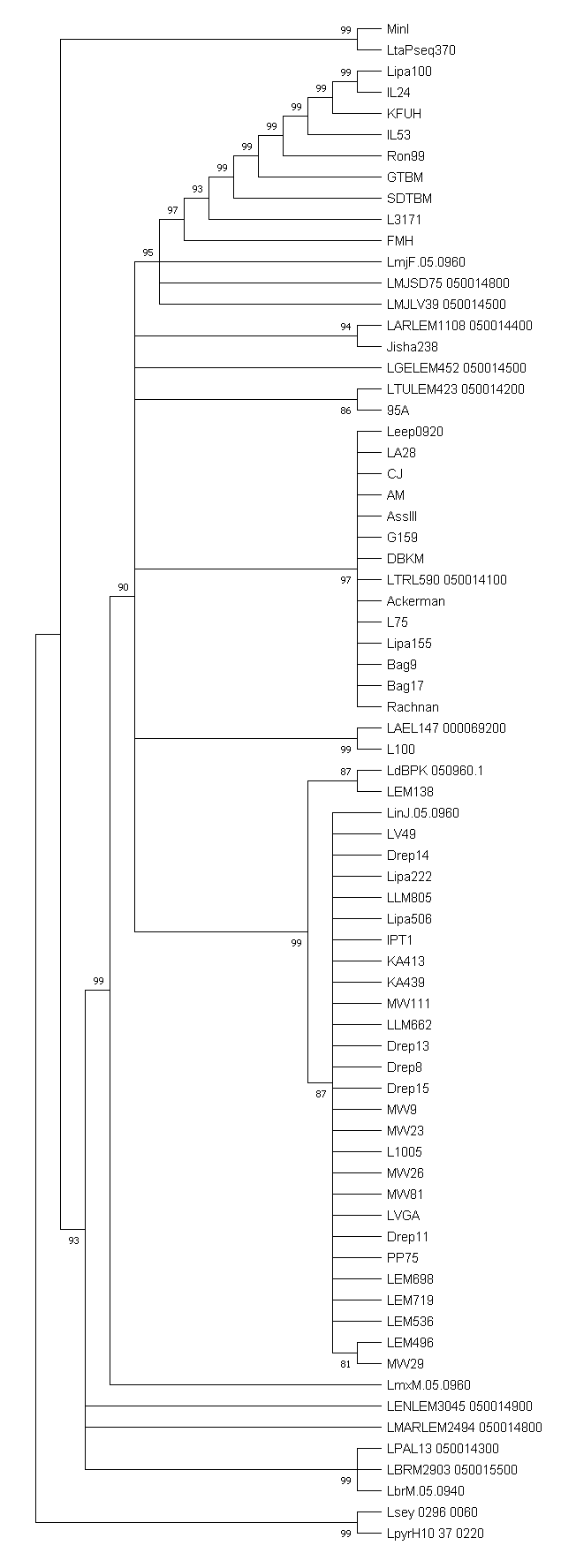

Supplement: S5 Fig — The bootstrap consensus tree inferred from 1000 replicates is taken to represent the evolutionary history of the taxa analyzed. Branches corresponding to partitions reproduced in less than 50% bootstrap replicates are collapsed. The percentage of replicate trees in which the associated taxa clustered together in the bootstrap test (1000 replicates) are shown next to the branches. The evolutionary distances were computed using the Tamura 3-parameter method and are in the units of the number of base substitutions per site. The rate variation among sites was modeled with a gamma distribution (shape parameter = 0.65). This analysis involved 72 nucleotide sequences. Codon positions included were 1st+2nd+3rd+Noncoding. All ambiguous positions were removed for each sequence pair (pairwise deletion option). There were a total of 662 positions in the final dataset. Evolutionary analyses were conducted in MEGA X. (TIF) [file pntd.0009530.s005.tif]

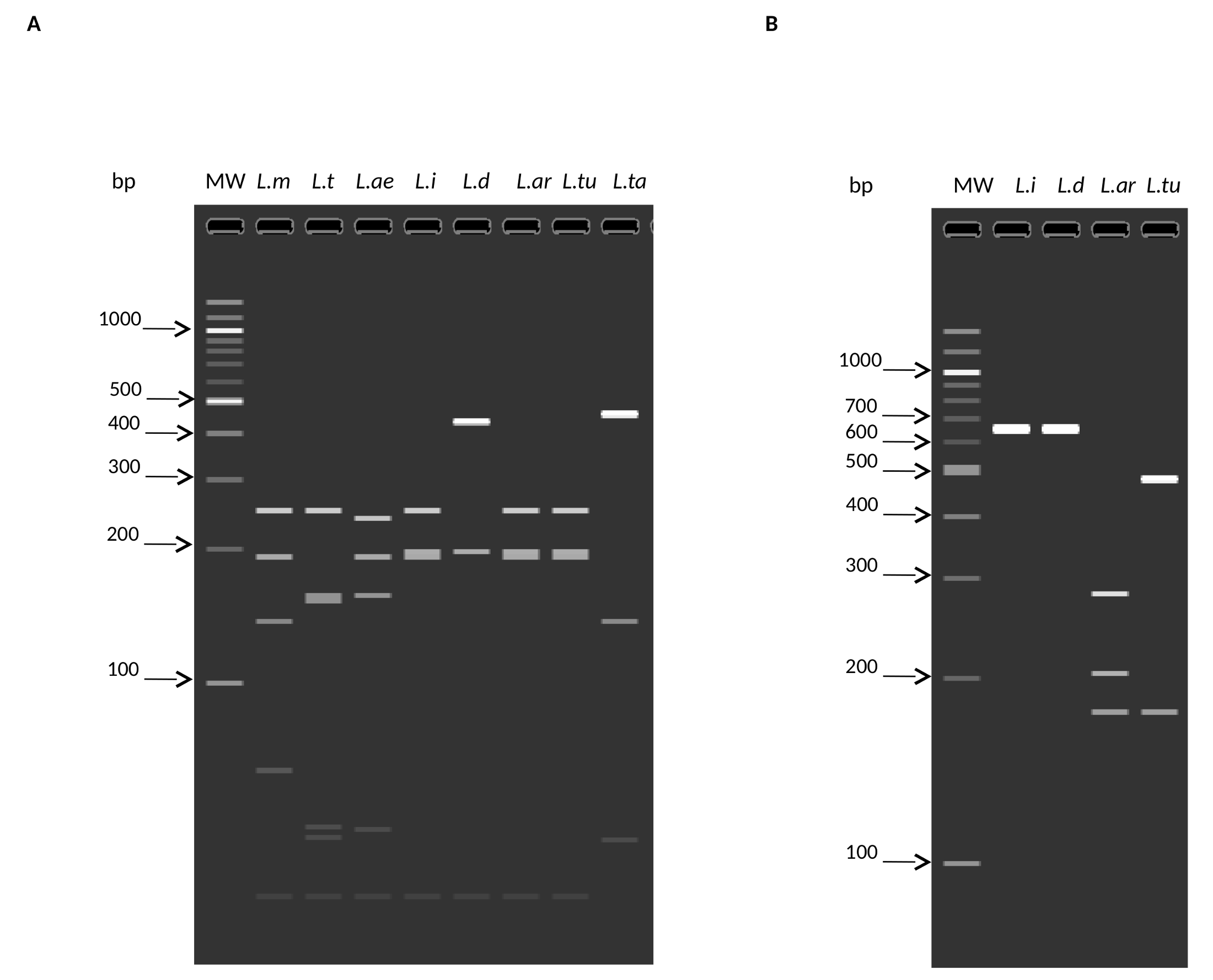

Supplement: S6 Fig — A. After double digestion with HaeIII&KpnI endonucleases. B. After double digestion with SacI and PvuII of the PCR products. The parasites that could not be distinguished by HaeIII/KpnI digestion are differentiated by the SacI/PvuII restriction. MW: 100 bp Molecular Weight. L.m: L. major, L.i: L. infantum, L.t: L. tropica, L.d: L. donovani, L.ae: L. aethiopica, L.ar: L. arabica, L.tu: L. turanica, L.ta: L. tarentolae. (TIF) [file pntd.0009530.s006.tif]

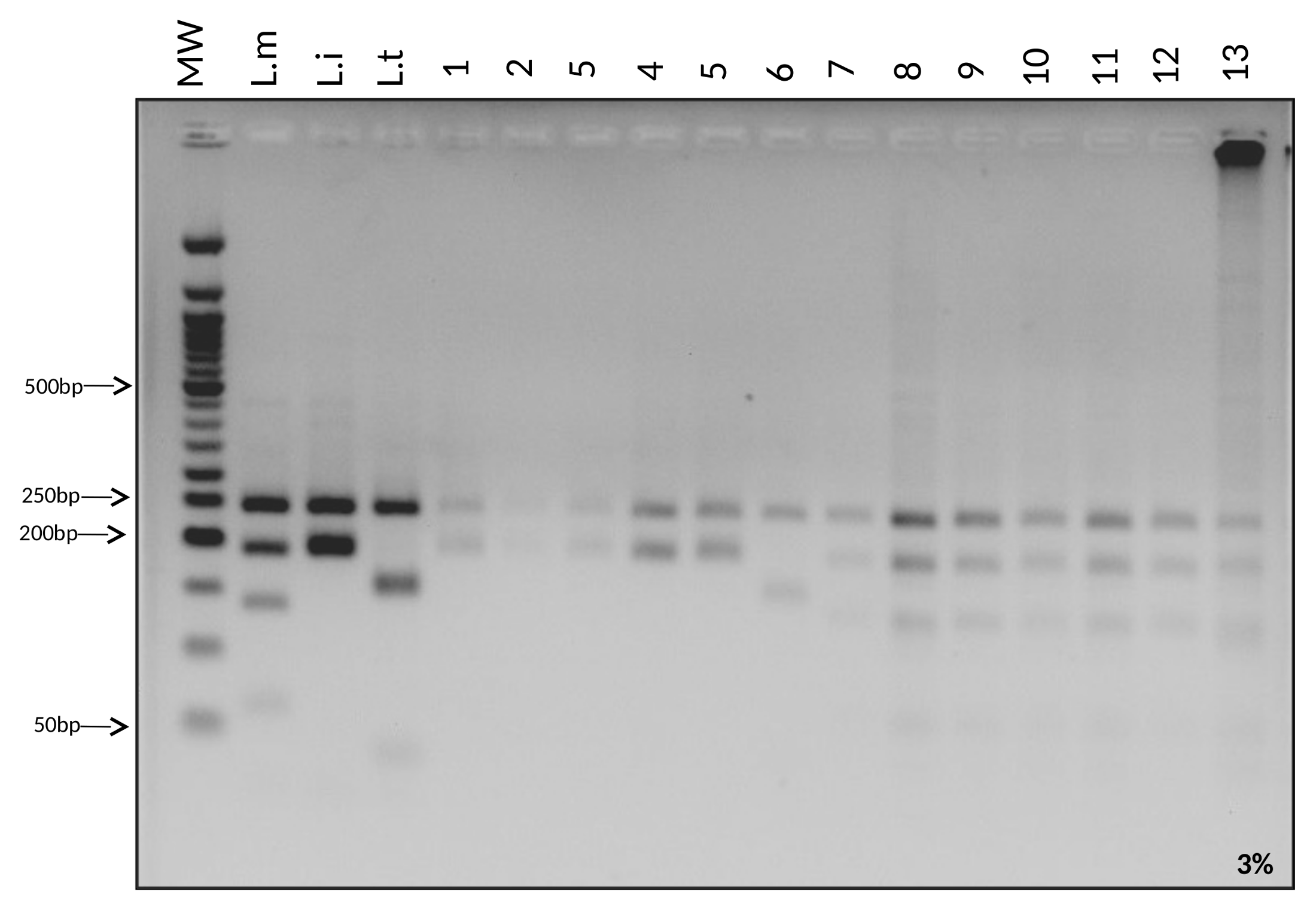

Supplement: S7 Fig — DNA was isolated directly from clinical samples, amplified by DPPIII-PCR and double digested with HaeIII-KpnI. MW: 50bp Molecular Weight. L. m: L. major (IL24), L. i: L. infantum (Drep 5) and L. t: L. tropica (LA28). Lanes 1–13: Clinical samples. (TIF) [file pntd.0009530.s007.tif]

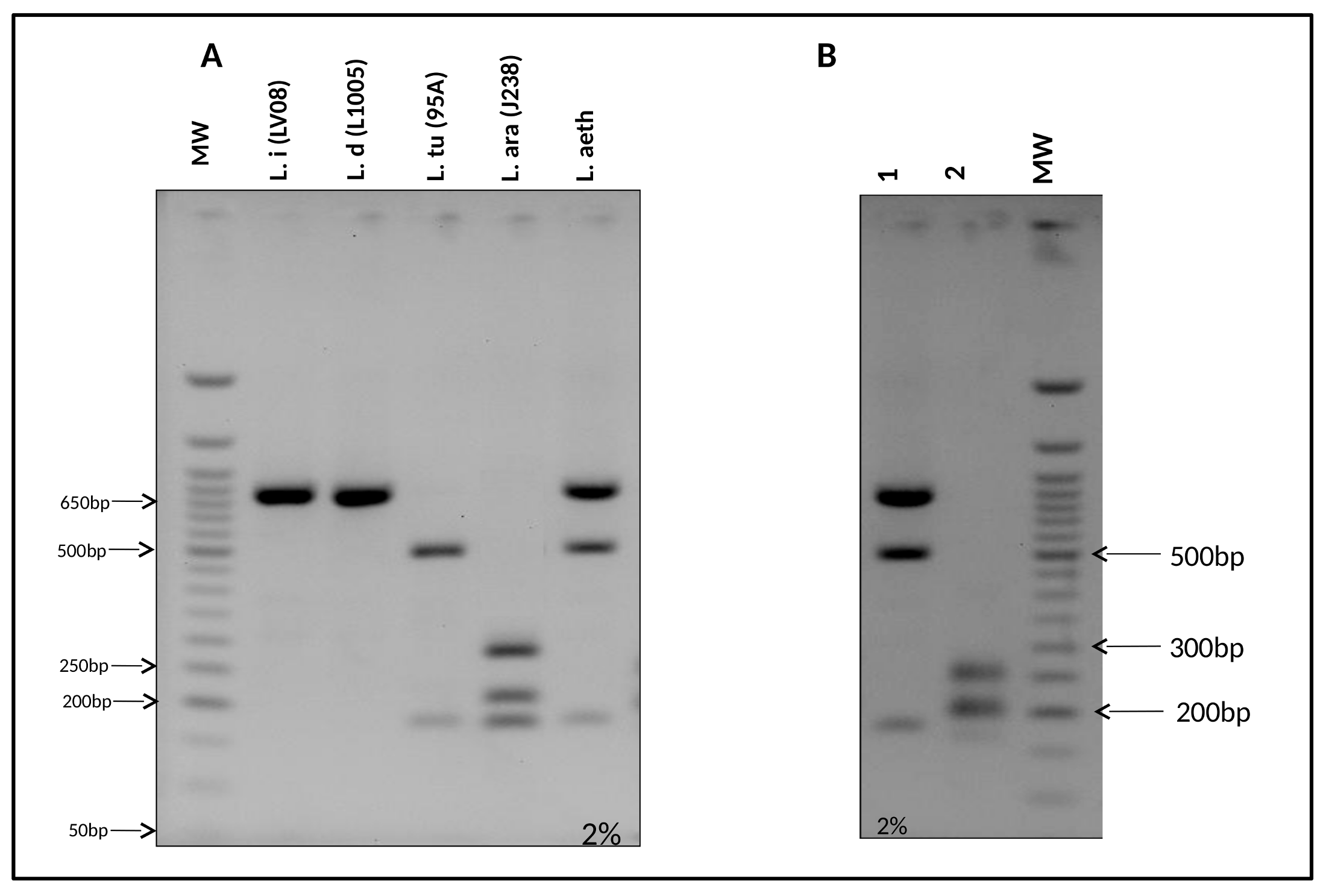

Supplement: S8 Fig — (A) Second round digestion of L. aethiopica strain. MW: 50bp Molecular weight; L. i: L. infantum (LV08); L. d: L. donovani (L1005); L. tu: L. turanica (95A); L. ara: L. arabica (J238); L. aeth: L. aethiopica (L100). (B) Two round digestion of L. aethiopica strain visualized on a 2% agarose gel. Lane 1: HaeII-KpnI double digestion, Lane 2: SacI-PvuII double digestion. (TIF) [file pntd.0009530.s008.tif]
