# Supplementary material for: Dipeptidyl peptidase III as a DNA marker to investigate epidemiology and taxonomy of Old World Leishmania species
Source: PLoS Negl Trop Dis. 2021 Jul 26;15(7):e0009530. doi: 10.1371/journal.pntd.0009530 (PMC8341715; doi:10.1371/journal.pntd.0009530)
Supplement: S1 Table — (DOCX) [file pntd.0009530.s009.docx]

**S1 Table. Selection of studied strains**

| **Code** | **WHO code** | **Origin** | **Pathology** | **Zymodeme** | **Species** |
| --- | --- | --- | --- | --- | --- |
| **Lipa100** | MHOM/DZ/09/LIPA100 | Algeria | CL | MON-25 | *L. major* |
| **SDTBM** | ISAL/IN/73/STDBM | India | NA | LON-6 | *L. major* |
| **GTBM** | MMER/IN/73/GTBM | India | NA | MON-23 | *L. major* |
| **L3171** | MHOM/IL/80/Friedlin | Israel | CL | MON-103 | *L. major* |
| **IL24** | MHOM/IL/83/IL24 | Israel | CL | MON-66 | *L. major* |
| **IL53** | MHOM/IL/83/IL53 | Israel | CL | MON-67 | *L. major* |
| **KFUH** | MHOM/SA/84/KFUH-7532 | Saudi Arabia | CL | LON-65 | *L. major* |
| **Ron99** | MPSA/TN/87/Ron99 | Tunisia | NA | MON-25 | *L. major* |
| **Ron155*** | MPSA/TN/ 87/Ron155 | Tunisia | NA | MON-25 | *L. major* |
| **FMH** | MHOM/TN/90/FMH | Tunisia | CL | ND | *L. major* |
| **K001*** | MHOM/AF/82/K001 | Afghanistan | CL | MON-58 | *L. tropica* |
| **Lipa155** | MHOM/SR/86/LIPA155 | Algeria | CL | MON-76 | *L. tropica* |
| **Ackerman** | MHOM/ASIA/74/Ackerman | Asia | - | - | *L. tropica* |
| **LA28** | MHOM/GR/00/LA28 | Greece | CL | LON-16 | *L. tropica* |
| **DBKM** | MCAN/IN/71/DBKM | India | NA | MON-62 | *L. tropica* |
| **Bag9** | MHOM/IQ/76/BAG9 | Iraq | CL | MON-53 | *L. tropica* |
| **Bag17** | MHOM/IQ/76/BAG17 | Iraq | CL | LON-24 | *L. tropica* |
| **L75** | MHOM/IQ/65/L75 | Iraq | CL | MON-6 | *L. tropica* |
| **G159** | MHOM/IL/00/Gabaï159 | Israel | CL | LON-9 | *L. tropica* |
| **Rachnan** | MHOM/IL/78/Rachnan | Israel | CL | MON-60 | *L. tropica* |
| **A sinaï III** | MHOM/IQ/73/A Sinaï III | Iraq | CL | LON-11 | *L. tropica* |
| **AM** | MHOM/TN/06/AM | Tunisia | CL | ND | *L. tropica* |
| **CJ** | MHOM/TN/06/CJ | Tunisia | CL | ND | *L. tropica* |
| **Leep0920**** | MHOM/TN/09/Leep0920 | Libya | CL | ND | *L. tropica* |
| **Lipa1153*** | MHOM/DZ/01/LIPA1153 | Algeria | CL | MON-24 | *L. infantum* |
| **Lipa506** | MHOM/DZ/96/LIPA506 | Algeria | CL | MON-24 | *L. infantum* |
| **Lipa222** | **-** | Algeria | VL | - | *L. infantum* |
| **MW111** | MHOM/SD/00/MW111 | Sudan | VL | MON-30 | *L. infantum* |
| **LLM805** | MCAN/ES/98/LLM805 | Spain | CanL | MON-1 | *L. infantum* |
| **LLM662** | MCAN/ES/97/LLM662 | Spain | VL | MON-1 | *L. infantum* |
| **IPT1** | MHOM/TN/80/IPT1 | Tunisia | VL | MON-1 | *L. infantum* |
| **LV50*** | MHOM/TN/94/LV50 | Tunisia | VL | MON-1 | *L. infantum* |
| **LV49** | MHOM/TN/94/LV49 | Tunisia | VL | MON-24 | *L. infantum* |
| **Drep14** | MHOM/TN/97/Drep14 | Tunisia | CL | MON-24 | *L. infantum* |
| **Drep05** | MHOM/TN/96/Drep05 | Tunisia | CL | MON-1 | *L. infantum* |
| **IPT1** | MHOM/TN/80/IPT1 | Tunisia | VL | MON-1 | *L. infantum* |
| **KA413** | MHOM/TN/87/KA 413 | Tunisia | VL | MON-1 | *L. infantum* |
| **KA439** | MHOM/TN/88/KA 439 | Tunisia | VL | MON-1 | *L. infantum* |
| **Drep11** | MHOM/TN/97/Drep11 | Tunisia | CL | MON-24 | *L. infantum* |
| **Drep13** | MHOM/TN/97/Drep13 | Tunisia | CL | MON-24 | *L. infantum* |
| **Drep08** | MHOM/TN/96/Drep08 | Tunisia | CL | MON-1 | *L. infantum* |
| **Drep15** | MHOM/TN/98/Drep15 | Tunisia | CL | MON-24 | *L. infantum* |
| **LVGA** | MHOM/TN/95/LVGA | Tunisia | VL | ND | *L. infantum* |
| **LEM698** | MHOM/ET/67/HU3 | Ethiopia | VL | MON-18 | *L. donovani* |
| **LEM138** | MHOM/IN/00/DEVI | India | VL | MON-2 | *L. donovani* |
| **LEM496** | MHOM/KE/75/H9 | Kuwait | VL | MON-32 | *L. donovani* |
| **LEM719** | IMRT/KE/62/LRC-L57 | Kuwait | - | MON-37 | *L. donovani* |
| **LEM536** | MHOM/SA/81/JEDDAH-KA | Saudi Arabia | VL | MON-31 | *L. donovani* |
| **MW29** | MHOM/SD/00/MW29 | Sudan | VL | MON-18 | *L. donovani* |
| **L1005^+^** | MHOM/ET/72/GEBRE1 | Ethiopia | VL | MON-82 | *L. donovani* |
| **MW09^+^** | MHOM/SD/00/MW09 | Sudan | VL | MON-82 | *L. donovani* |
| **MW23^+^** | MHOM/SD/00/MW23 | Sudan | VL | MON-82 | *L. donovani* |
| **MW26^+^** | MHOM/SD/00/MW26 | Sudan | PKDL | MON-82 | *L. donovani* |
| **MW81^+^** | MHOM/SD/00/MW81 | Sudan | VL | MON-82 | *L. donovani* |
| **PP75^++^** | MHOM/BR/1974/PP75 | Brazil | VL | MON-1 | *L. infantum* |
| **95A** | MRHO/SU/74/95A | Sudan | CL | MON-64 | *L. turanica* |
| **Jisha238** | MPSA/SA/84/Jisha238 | Saudi Arabia | NA | MON-64 | *L. arabica* |
| **L100** | MHOM/ET/72/L100 | Ethiopia | CL | MON-14 | *L. aethiopica* |
| **Min I** | IMIN/IT/86/MIN1 | Italy | NA | - | *L. tarentolae* |

*: not sequenced PCR product

**: the strain was isolated in Tunisia

^+^: East African *L. donovani* strains (also known as *L. archibaldi*)

^++^: Brazilian*L. infantum* strain (also known as.*L. chagasi*)

ND: Not Determined

NA: Not applicable

- : Unknown
