# Supplementary material for: Dipeptidyl peptidase III as a DNA marker to investigate epidemiology and taxonomy of Old World Leishmania species
Source: PLoS Negl Trop Dis. 2021 Jul 26;15(7):e0009530. doi: 10.1371/journal.pntd.0009530 (PMC8341715; doi:10.1371/journal.pntd.0009530)
Supplement: S3 Table — (DOCX) [file pntd.0009530.s011.docx]

**S3 Table. Selection of 19 gene sequences taken from the public TritrypDB databases (Release 26 (14 Oct 15) and used for the typing scheme elaboration**

| **Organism** | **Geographic Origin** | **Strain** | **Gene ID** | **Chromosome** | **Genomic location (strand)** |
| --- | --- | --- | --- | --- | --- |
| ***L. seymouri*** | USA (Florida) | ATCC 30220 | Lsey_0296_0060 | 5 | 21,721 - 23,766 (+) |
| ***L. pyrrhocoris*** | - | H10 | LpyrH10_37_0220 | 5 | 78,062 - 80,779 (+) |
| ***L. aethiopica*** | Ethiopia | MHOM/ET/72/L100 | LAEL147_000069200 | 5 | 402,256 - 404,295 (+) |
| ***L. arabica*** | - | LEM1108 | LARLEM1108_050014400 | 5 | 347,858 - 349,897 (+) |
| ***L. braziliensis*** | Brazil | MHOM/BR/75/M2904 | LbrM.05.0940 | 5 | 345,257 - 347,296 (+) |
| ***L. braziliensis*** | Brazil | MHOM/BR/75/M2903 | LBRM2903_050015500 | 5 | 390,722 - 392,761 (+) |
| ***L. donovani*** | Nepal | BPK282A1 | LdBPK_050960.1 | 5 | 356,842 - 358,881 (+) |
| ***L. enriettii*** | Brazil | LEM3045 | LENLEM3045_050014900 | 5 | 347,323 - 349,362 (+) |
| ***L. gerbilli*** | China | LEM452 | LGELEM452_050014500 | 5 | 351,107 -353,146 (+) |
| ***L. infantum*** | Spain | JPCM5 | LinJ.05.0960 | 5 | 354,919 - 356,958 (+) |
| ***L. sp. MAR*** | Martinique | LEM2494 | LMARLEM2494_050014800 | 5 | 341,317 - 343,356 (+) |
| ***L. major*** | Israel | Friedlin | LmjF.05.0960 | 5 | 356,672 - 358,711 (+) |
| ***L. major*** | USSR | LV39c5 | LMJLV39_050014500 | 5 | 353,266 - 355,305 (+) |
| ***L. major*** | Senegal | SD 75.1 | LMJSD75_050014800 | 5 | 348,964 - 351,003 (+) |
| ***L. mexicana*** | Guatemala | MHOM/GT/01/U1103 | LmxM.05.0960 | 5 | 358,745 - 360,784 (+) |
| ***L. panamensis*** | Colombia | MHOM/COL/81/L13 | LPAL13_050014300 | 5 | 327,336 - 329,375 (+) |
| \| ***L. tropica*** \| \| --- \| | Israel | MHOM/IL/90/LRC-L590 | LTRL590_050014100 | 5 | 363,658 - 365,697 (+) |
| ***L. turanica*** | USSR | LEM423 | LTULEM423_050014200 | 5 | 376,139 - 378,178 (+) |
| ***L. tarentolae*** | Italy | Parrot-TarII | LtaP05.1040 | 5 | 355,985 - 356,455 (+) |
